# Heterophyllin B: Combining Isotropic and Anisotropic NMR for the Conformational Analysis of a Natural Occurring Cyclic Peptide

**DOI:** 10.1002/mrc.5523

**Published:** 2025-04-20

**Authors:** Anton F. Ketzel, Yang Hu, Xiao‐Lu Li, Jiaqian Li, Xinxiang Lei, Han Sun

**Affiliations:** ^1^ Research Unit of Structural Chemistry & Computational Biophysics Leibniz‐Forschungsinstitut für Molekulare Pharmakologie (FMP) Berlin Germany; ^2^ Strukturelle Chemische Biologie und Cheminformatik, Institut für Chemie Technische Universität Berlin Berlin Germany; ^3^ Institute of Medical Science The Second Hospital of Shangdong University Jinan China; ^4^ School of Pharmaceutical Sciences South Central University for Nationalities Wuhan China; ^5^ State Key Laboratory of Applied Organic Chemistry, Lanzhou Magnetic Resonance Center, College of Chemistry and Chemical Engineering Lanzhou University Lanzhou China

**Keywords:** anisotropic NMR, CENSO, conformational analysis, CREST, cyclic peptide, DFT, NMR, RCSA, RDC

## Abstract

Heterophyllin B is a natural occurring cyclic peptide with diverse attributed bioactivities. NMR‐based conformational analysis of cyclic peptides often poses a challenge due to limited isotropic solution‐state NMR data. In this study, we combined isotropic and anisotropic NMR observables including *J*‐coupling, NOEs, amide proton temperature coefficients, and residual dipolar couplings (RDCs), which enabled the determination of a minimal conformational ensemble of heterophyllin B in methanol at density functional theory (DFT) accuracy. For conformational sampling of a cyclic peptide with a high degree of conformational freedom, we proposed a computational strategy that combines the Conformer–Rotamer Ensemble Sampling Tool (CREST) with the Commandline Energetic SOrting (CENSO). This combined computational and NMR‐based approach offers a robust framework for the conformational analysis of cyclic peptides.

## Graphical abstract

A minimal conformational ensemble of cyclic peptide heterophyllin B is presented at the density functional theory accuracy using isotropic and anisotropic NMR.

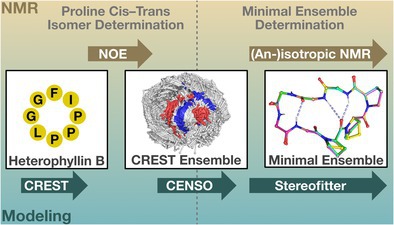

## 1 Introduction

Cyclic peptides have garnered increasing attention in drug development due to their unique potential for targeting traditionally “undruggable” targets, such as protein–protein interactions [1, 2, 3]. These molecules have been developed to address a highly diverse spectrum of diseases, including cancer, various infectious diseases, endocrine and metabolic disorders, as well as cardiovascular disorders [1]. Compared with their linear counterparts, cyclic peptides are often more stable, thanks to their cyclic structure, which resists enzymatic degradation [4]. Additionally, they often exhibit enhanced membrane permeability, resulting in improved oral bioavailability [5, 6]. Currently, 40 cyclic peptide drugs are approved for therapeutic use, with the majority derived from natural sources [2, 3].

For structure‐based drug development, determining the three‐dimensional structures of cyclic peptides in solution is essential [7]. Furthermore, establishing these structures in both polar and apolar solvents is also crucial for understanding the cell permeability of cyclic peptides [8, 9]. For example, a recent study demonstrated the use of NMR‐determined structures of a large number of cyclic peptides, combined with computational design, to investigate the principles of membrane permeability of cyclic peptides. This approach further enabled the design of several new cyclic peptides with improved cell permeability in vitro and enhanced oral bioavailability in vivo [10].

The state‐of‐the‐art methods for determining the three‐dimensional structure of cyclic peptides are X‐ray crystallography and NMR spectroscopy [11]. The latter primarily relies on isotropic NMR structural restraints, such as nuclear Overhauser effects (NOEs), scalar coupling (*J*‐coupling), and amide proton temperature coefficients [12]. Parameters such as amide proton temperature coefficient are especially useful for evaluating the formation of intramolecular hydrogen bonds [13, 14]. However, quite often, accurately determining the structures of cyclic peptides remains a significant challenge, especially when isotropic NMR restraints are sparse. In such cases, several studies have demonstrated the power of incorporating anisotropic NMR parameters such as residual dipolar couplings (RDCs) to improve structural determination of cyclic peptides [8, 15, 16, 17, 18, 19, 20, 21, 22].

In this study, we performed an integrated analysis by combining isotropic and anisotropic NMR data to determine the structure of a naturally occurring cyclic peptide, heterophyllin B (**1**). This cyclic octapeptide was originally extracted from the roots of the *Pseudostellaria heterophylla*, a plant commonly found in southern China and widely used in traditional Chinese medicine [23]. Heterophyllin B is cell‐permeable [24] and exhibits intriguing bioactive properties with therapeutic potential across a wide range of diseases [25, 26, 27, 28, 29].

Despite its promising characteristics, its conformational state has yet to be elucidated, which is crucial for accurate computational applications, such as molecular docking studies to understand its mode of actions at the atomistic scale.

We measured here an extensive set of isotropic and anisotropic NMR data, including NOEs, *J*‐couplings, amide proton temperature coefficients, RDCs, and residual chemical shift anisotropies (RCSAs). We propose an automated conformational sampling and selection strategy to generate a conformational ensemble, which is further reduced based on the anisotropic NMR data and ultimately validated against isotropic NMR parameters. Using this combined approach, we successfully determined a minimal conformational ensemble of **1** with density functional theory (DFT) accuracy.

## 2 Results and Discussion

### 2.1 Assignment of ^1^H and ^13^C NMR Signals for Heterophyllin B (**1**)

**FIGURE 1:**
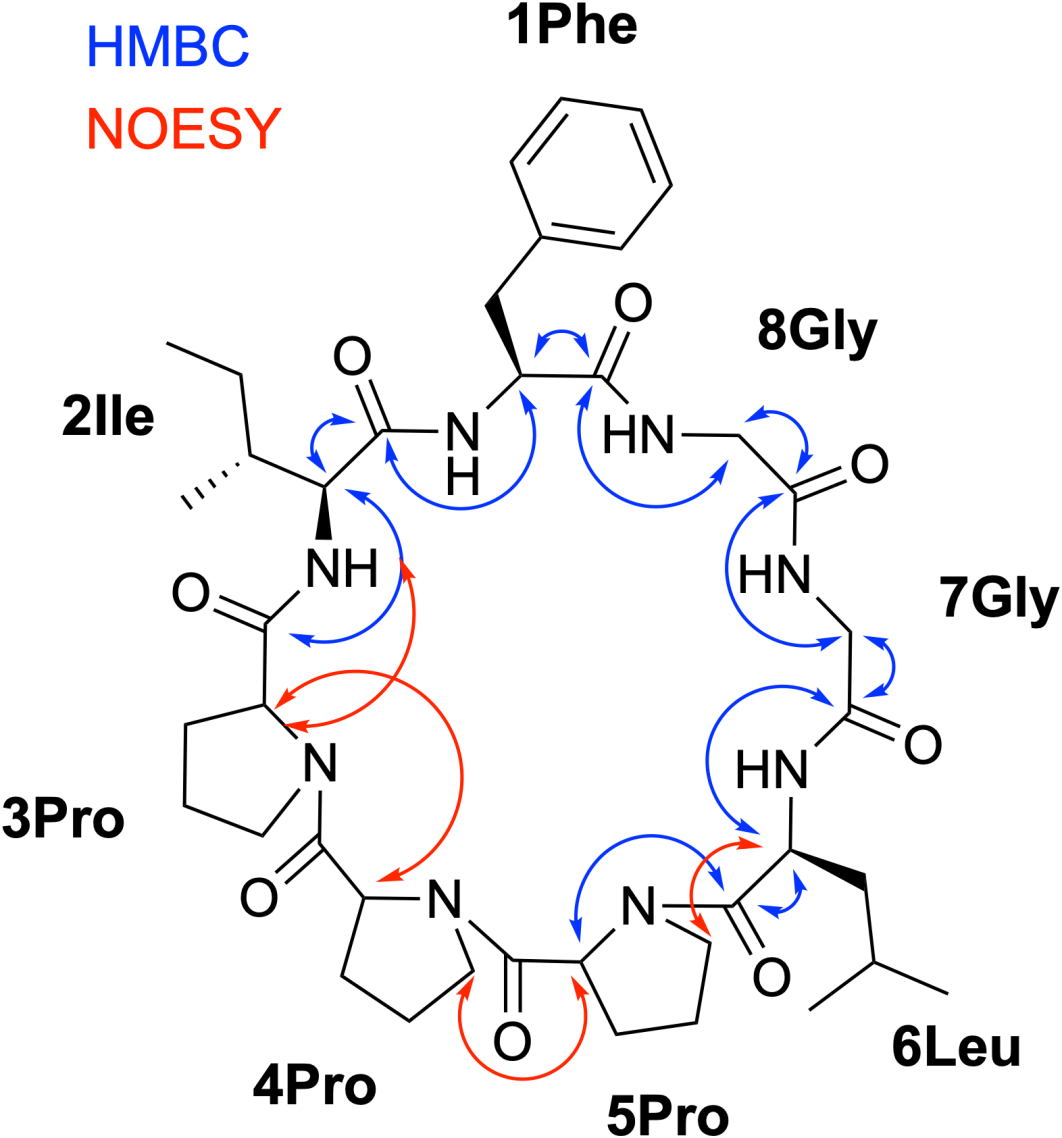
Chemical structure of heterophyllin B (**1**) with indicated amino acid residues and key HMBC and NOESY correlations for the assignment of the backbone.

^1^H and ^13^C NMR assignments of **1** in MeOD‐*d*_4_ were performed using standard 1D and 2D NMR experiments, including 1D‐^1^H, 1D‐^13^C, ^13^C,^1^H‐HSQC, ^13^C,^1^H‐HMBC, ^1^H,^1^H‐COSY, ^1^H,^1^H‐TOCSY, and ^1^H,^1^H‐NOESY (Table S1). Key HMBC and NOESY correlations of the backbone of **1** are indicated in Figure 1. Diastereotopic hydrogen signals have been assigned only as pairs and not individually.

### 2.2 Measurement of Isotropic and Anisotropic NMR Restraints for Heterophyllin B (**1**)

For the conformational determination of compound **1** (Figure 1), we measured isotropic structural parameters, including ^3^ *J*_HH_‐couplings (Table S1), ^1^H‐^1^H NOE correlations translated into distances (Table S2), and amide proton coefficients (Figure S1), as well as anisotropic NMR data including ^1^ *D*_CH_ RDCs and ^13^C‐ΔΔRCSAs (Table S3).

Details of the isotropic NMR experiments are provided in the Supporting Information. RDCs were measured by calculating the differences between one‐bond proton‐carbon couplings extracted from F2‐coupled ^1^H,^13^C‐CLIP‐HSQC spectra under isotropic and anisotropic conditions (Figure S2). It should be noted that methylene RDCs have been used in the subsequent analysis only as averaged values and not individually. For measuring ^13^C‐ΔΔRCSAs, ^13^C‐spectra of the initial and final alignment stages were recorded as described in our previous publications [30]. To measure ΔΔRCSAs, the liquid‐crystal‐forming oligopeptide AAKLVFF [31] was used in methanol to gradually form the anisotropic state. With high confidence, 10 backbone RDCs and eight backbone ΔΔRCSAs were measured (Table S3). Additionally, 14 RDCs were measured for the proline sidechains with sufficient accuracy. For the ΔΔRCSA measurements, the C_α_ atom of the 1Phe residue was chosen as the reference atom, as it has the smallest chemical shift anisotropy constant among all backbone atoms based on GIAO‐DFT calculations.

### 2.3 Determination of the Backbone Proline Conformation

**FIGURE 2:**
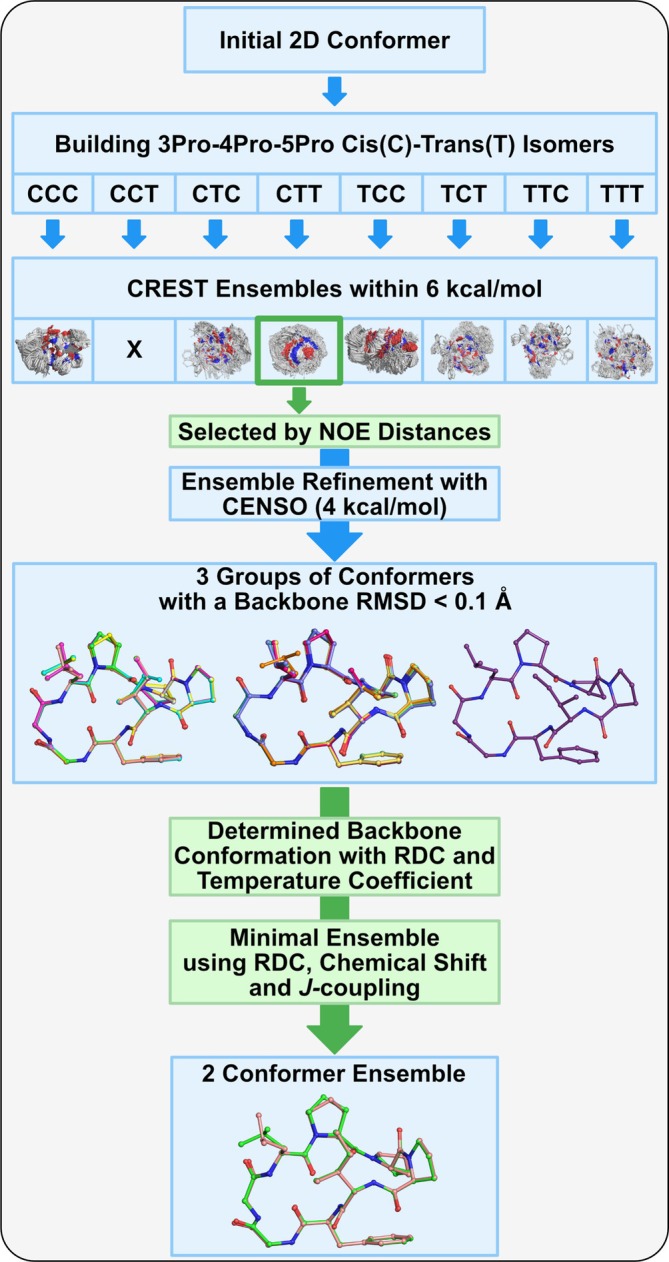
Workflow for the conformational analysis of **1** with computational steps being highlighted in blue and selection by experimental data in green. The structural ensemble has been marked with “X” when the backbone conformation was not stable in the sampling and converted to another conformation.

We employed a cross‐validation approach to generate a conformational ensemble that is fitted to and selected based on NMR restraints. Consequently, a pre‐generated ensemble obtained by computational methods is required. As illustrated in Figure 2, we performed a conformational sampling procedure in this study using the all‐atom force field GFN‐FF [32] in conjunction with the Conformer–Rotamer Ensemble Sampling Tool (CREST) [33, 34]. In this approach, possible conformers are generated through short meta‐dynamic simulations followed by optimization at the tight‐binding (xTB‐GFN2) [35] level of theory. The final ensembles from these methods includes all conformations within an energy window of 6 kcal/mol.

Because *cis*‐*trans* proline isomerization is a very slow interconversion process [36], reliably sampling all conformations using the above‐described computational approach is challenging. As compound **1** consists of three consecutive proline residues, we instead performed individual samplings for all possible combinations of *cis*/*trans* isomers of the proline amide bonds. From the samplings of the different starting structures, we observed that some CREST ensembles either did not change or only rarely altered the proline backbone conformation, while in others, the conformations quickly converted to one of the more stable conformations during the calculations. For two of these unstable ensembles, constrained sampling gave a better starting structure, yielding stable ensembles in subsequent unconstrained samplings. The obtained dihedrals of the three prolines for all *cis*/*trans* isomers are shown in Figures S3–S10. As a result, seven stable proline backbone conformations (3C‐4C‐5C, 3C‐4T‐5C, 3C‐4T‐5T, 3T‐4C‐5C, 3T‐4C‐5T, 3T‐4T‐5C, and 3T‐4T‐5T) were identified, each yielding ensembles of approximately 600–2000 conformers.

Because only one set of NMR signals was observed for compound **1**, we expect a single defined backbone conformation of the three prolines. From the analysis of the ^1^H,^1^H‐NOESY spectrum (see Table S2), three key distances for the proline residues were identified. These experimental distances were compared with the average distances calculated across the entire CREST ensemble for each stable conformation, using the mean absolute deviation (MAD) as the metric. As shown in Table 1, only the ensemble corresponding to 3C‐4T‐5T matches all three distances, exhibiting very small MAD. Therefore, we conclude that the backbone conformation of the three proline residues is 3C‐4T‐5T.

**TABLE 1:** NOE‐derived distances of three key hydrogen–hydrogen distances that are used to define the backbone proline conformation. Green indicates the backbone conformation with the lowest MAD compared with the experimental distances.

	Distances in [Å]			
Proline conformation	3H_α_‐4H_α_	5H_α_‐4H_δ1/2_	6H_α_‐5H_δ1_ ^a^	MAD
Exp.	2.18	2.22	2.16	—
3T‐4T‐5T	4.46	2.93	3.54	1.5
3T‐4T‐5C	4.60	2.64	4.51	1.7
3T‐4C‐5T	4.46	4.53	2.31	1.6
3T‐4C‐5C	4.44	4.55	4.51	2.3
3C‐4T‐5C	2.45	2.62	4.68	1.1
3C‐4C‐5C	2.54	4.55	4.56	1.7
3C‐4T‐5T	2.37	2.47	2.40	0.2

^a^ The shorter distance between the two methylene protons was selected without an unambiguous assignment.

### 2.4 Determination of the Peptide‐Backbone Conformation

**FIGURE 3:**
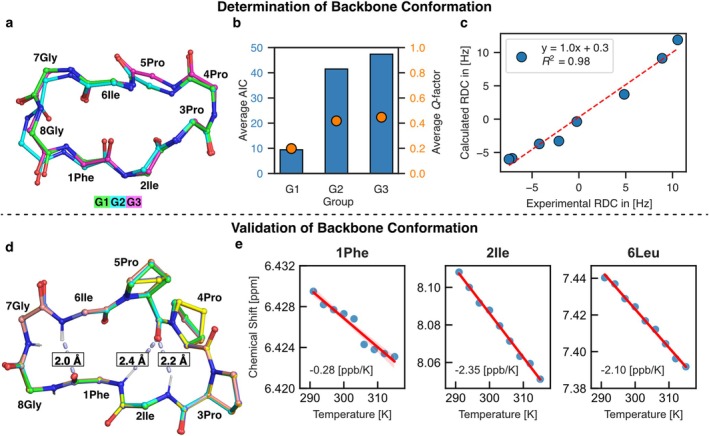
(a) Superimposed backbone conformations of groups G1, G2, and G3 without side chains. (b) AIC and *Q*‐factor from the RDC analysis using only 10 backbone RDC values. The resulting values are averaged across all conformers within each groups. (c) Linear regression of the experimental RDCs against the back‐calculated RDCs for the conformer of G1 with the lowest AIC value. (d) Six superimposed conformers of G1 with internal hydrogen bonds indicated. (e) Linear regression of the chemical shifts of the amide protons (1Phe, 2Ile, and 6Leu) with temperature, showing the amide temperature coefficient in [ppb/K]. Additional plots for the two glycine residues are shown in Figure S1.

After determining the proline backbone conformation, the 3C‐4T‐5T ensemble identified by CREST was refined and optimized using modern DFT methods, specifically PW6B95‐D4/def2‐TZVP/SMD (methanol) [37, 38, 39, 40], within the Commandline Energetic SOrting (CENSO) [41] approach. Based on Gibbs free energy using the modified rigid‐rotor‐harmonic‐oscillator (mRRHO) approximation [42], a final ensemble consisting of 13 conformations was generated. These conformations collectively represent 95% of the conformational ensemble at room temperature based on DFT free energies, the free energies of all DFT optimized conformers are shown in Table S4. The conformers were subsequently subjected to further GIAO‐NMR shielding and coupling constant calculations at the PW6B95/pcSseg‐2 [43]/SMD (methanol) level of theory. Chemical shieldings have been converted to chemical shifts using the linear scaling parameters obtained by fitting a small set of experimental chemical shifts (Figure S11) [44, 45]. Using a root mean square deviation (RMSD) filter on the backbone (Table S5), three conformational groups were identified (Figure 3a). Groups G1 and G2 each contain six conformers and are overall similar; however, a peptide bond plane flip between 7Gly and 8Gly was observed when comparing the two groups.

The third group, G3, consists of a single conformer, which represents only a minor deviation from G1 but has a slightly larger RMSD of 0.1. By utilizing only the C_α_–H_α_ RDCs of the backbone in Stereofitter [46, 47], as shown in Figure 3b, the average *Q‐*factor [48] and AIC [49] value for G1 are noticeably lower compared with the other two groups. When applying the same RDC data in a multi‐conformer single‐tensor (MCST) procedure, a single conformer from G1 still exhibits the lowest AIC, even when compared with ensembles containing up to three conformers (details provided in the Tables S6–S8). Therefore, we propose that the backbone conformation of **1** consists of a single backbone conformer in methanol.

We further used backbone ΔΔRCSA data to distinguish between the three conformations, G1–G3. Again, G1 exhibited, on average, a lower *Q‐*factor and AIC compared with G2 and G3 (details provided in Tables S9–S10). However, the ability to discriminate using ΔΔRCSAs was significantly lower compared with RDCs and, therefore, it was not used in the subsequent analysis. It is worth noting that combining RDC and ΔΔRCSA in the single value decomposition (SVD) procedure, as implemented in Stereofitter, worsened the fitting performance compared with using only a single type of data (Tables S11 and S12).

To further validate the determined backbone conformation, the internal hydrogen‐bonding pattern was examined using amide proton temperature coefficients obtained from variable‐temperature NMR experiments (Figure 3e). For this purpose, 1D‐^1^H‐spectra were measured in methanol at 3 K intervals between 291 and 315 K. By performing linear regression of the amide proton chemical shifts against the temperature, the temperature coefficients were determined as the slope (Figure 3e). Comparison of these coefficients with empirical data [50] indicates that internal hydrogen bonds involving 1Phe, 2Ile, and 3Leu are very likely, whereas hydrogen bonds involving 7Gly and 8Gly are unlikely. This observation agrees well with the conformers of G1, as shown in Figure 3d, where the shortest hydrogen–oxygen distances are indicated.

Although in the current study, C‐H RDCs in combination with isotropic NMR data were sufficient to define the main conformational state of heterophyllin B reliably, we acknowledge that additional experimental data such as N‐H RDCs could provide further experimental restraints and thereby increase confidence in the determined structure.

### 2.5 Determination of Minimal Ensemble

**FIGURE 4:**
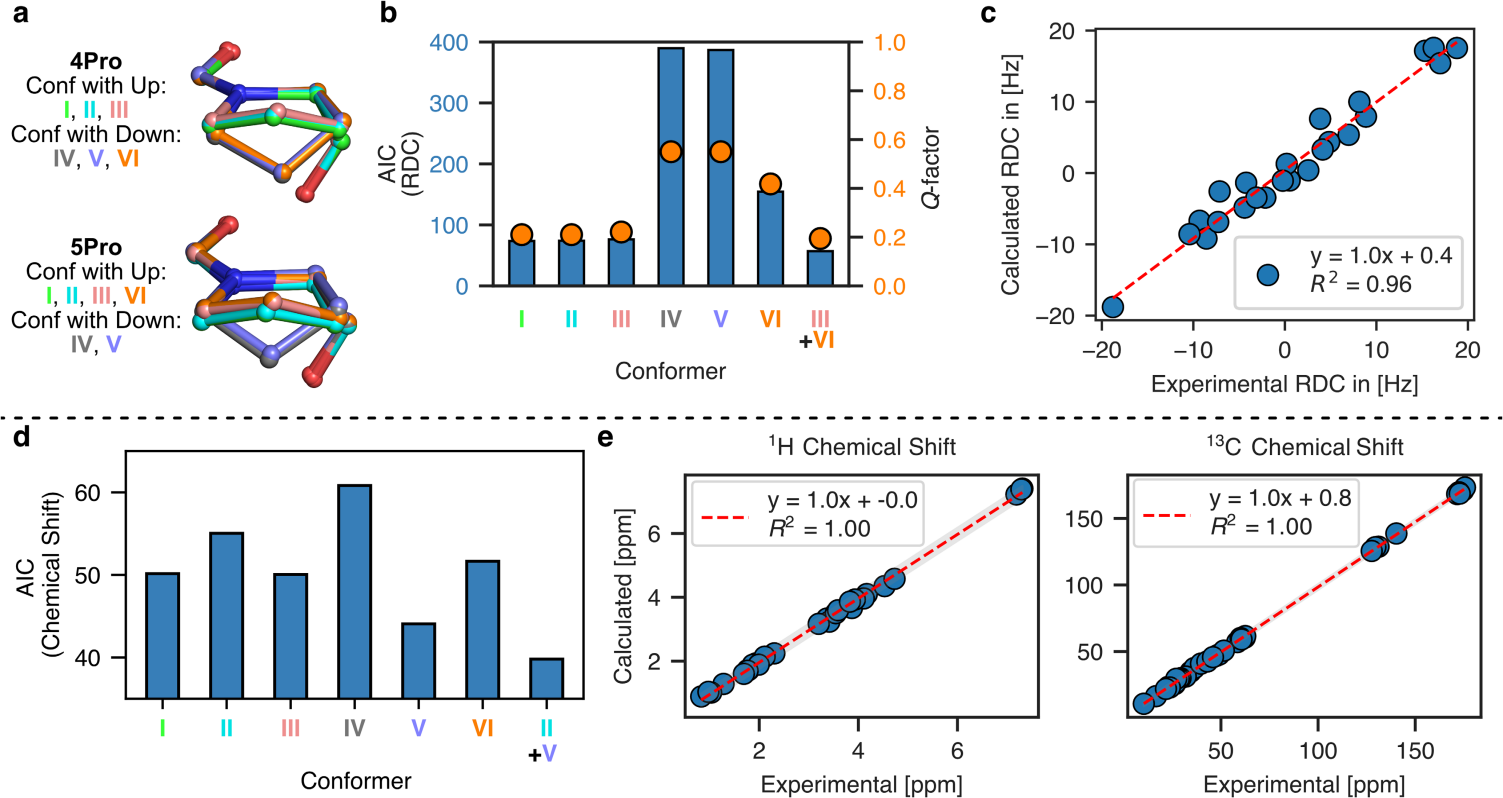
(a) Superimposed structures of 4Pro and 5Pro for the six conformers included in the backbone group G1, with the proline ring conformation indicated for each conformer. (b) AIC and *Q‐*factor from the RDC analysis using 24 backbone and proline RDCs. In addition to single conformers, the multiple‐conformer ensemble with the lowest AIC is shown. (c) Linear regression of the experimental RDCs against the calculated RDCs for the ensemble (**III** + **VI**). (d) AIC values and (e) linear regression comparing the experimental and DFT calculated chemical shifts at the PW6B95/pcSseg‐2/SMD (methanol) level of theory.

At this stage, an ensemble of six conformers remained, all of which yielded similar results when fitted against NOEs, backbone RDCs, and amide proton temperature coefficients, as discussed above. While the side chains of 1Phe, 2Ile, and 6Leu are expected to be flexible, the proline‐ring conformation is more defined [51]. For 3Pro, which was determined to have a *cis* amide conformation, the pyrrolidine ring was consistent across all six conformers. However, for 4Pro and 5Pro, the conformers differed in their pyrrolidine conformations. To better define the conformation of these prolines, the sidechain RDCs were incorporated into the SVD fitting (see for details Tables S13–S15). As shown in Figure 4b, single conformers, most notably conformers **I, II,** and **III**, still yielded quite reasonable results with *Q‐*factors around 0.22. However, the best results were obtained with an ensemble of two conformers (**III** + **VI**), which gave a *Q‐*factor of 0.19. In this ensemble conformer **III** was weighted at 73% and conformer **VI** with 27%. Interestingly, both conformers in this ensemble shared the same 5Pro conformation but differ in their 4Pro conformations. This suggests that 4Pro undergoes fast exchange between two possible conformations, whereas 3Pro and 5Pro are structurally more restricted.

To further elucidate this, we compared the experimental ^1^H and ^13^C chemical shifts with DFT calculated shifts. Based on AIC values, the discrimination between different conformers was less pronounced compared with the RDC analysis. Nevertheless, conformer **V** yielded the lowest single‐conformer AIC of 44 (Table S16). Notably, as observed with the RDC, using a conformational ensemble (**II** + **V**) resulted in an even lower AIC value (40). It is worth mentioning that similarly low AIC, around 43–44, were obtained when substituting conformer **II** with **I** or **III** (Table S17).

While the results for 5Pro remain somewhat ambiguous, 3Pro and 4Pro were both well described by the identified conformational ensembles. Overall, the RDC data provided better discrimination between conformers compared with the chemical shifts. Finally, the calculated *J‐*coupling constants have been compared with the experimentally determined ones. All six conformers showed good agreement with the experimental data (Tables S18 and S19). It should be noted, however, that only four coupling constants, primarily involving backbone hydrogens, were unambiguously determined from the ^1^H spectrum.

## 3 Conclusion

The backbone conformation of the natural occurring cyclic peptide heterophyllin B in methanol was determined with high confidence. Both isotropic and anisotropic NMR observables were used and consistently supported a single backbone conformation. Additionally, the side chain conformation of three proline residues was elucidated, revealing that only an ensemble of two conformers could adequately describe the experimental data.

## Conflicts of Interest

Han Sun is a guest editor of the special issue “Anisotropic NMR spectroscopy” of *Magnetic Resonance in Chemistry*. In accordance with the journal's policy and COPE guidelines, the manuscript was handled by an independent editor to ensure a fair and unbiased review process.

### Peer Review

The peer review history for this article is available at https://www.webofscience.com/api/gateway/wos/peer‐review/10.1002/mrc.5523.

## Supporting information


**Data S1.** The Supporting Information contain experimental procedures, computational details, plotted NMR spectra (Figures S12–S22), and Cartesian coordinates (Tables S20 and S21) for the determined minimal ensemble.
Additionally, the output files from CREST, CENSO, and ORCA and the original NMR data are deposited at https://doi.org/10.5281/zenodo.14699304.

## Data Availability

The data that support the findings of this study are openly available in Zenodo at https://zenodo.org/records/14699304, reference number 14699304.
