# Supplementary material for: Heterophyllin B: Combining Isotropic and Anisotropic NMR for the Conformational Analysis of a Natural Occurring Cyclic Peptide
Source: Magn Reson Chem. 2025 Apr 20;63(5-6):417–23. doi: 10.1002/mrc.5523 (PMC12053296; doi:10.1002/mrc.5523)
Supplement: Supplementary file 1 — Data S1. The Supporting Information contain experimental procedures, computational details, plotted NMR spectra (Figures S12–S22), and Cartesian coordinates (Tables S20 and S21) for the determined minimal ensemble. Additionally, the output files from CREST, CENSO, and ORCA and the original NMR data are deposited at https://doi.org/10.5281/zenodo.14699304. [file MRC-63-417-s001.docx]

Supplementary Information

Heterophyllin B: Combining Isotropic and Anisotropic NMR for the Conformational Analysis of a Natural Occurring Cyclic Peptide

Anton F. Ketzel, ^[a, b]^ Yang Hu, ^[a, b]^ Xiao-Lu Li,^[a, c]^ Jiaqian Li,^[d]^ Xinxiang Lei,^[d, e]^ Han Sun*^[a, b]^

[a] Research Unit of Structural Chemistry & Computational Biophysics,
Leibniz-Forschungsinstitut für Molekulare Pharmakologie (FMP)

13125 Berlin, Germany
E-mail: hsun@fmp-berlin.de

[b] Institut für Chemie, Strukturelle Chemische Biologie und Cheminformatik,
Technische Universität Berlin

10623 Berlin, Germany

[c] Institute of Medical Science,

The Second Hospital of Shandong University

250033 Jinan, China

[d] School of Pharmaceutical Sciences,

South Central University of Nationalities

430074 Wuhan, China

[e] State Key Laboratory of Applied Organic Chemistry,

Lanzhou Magnetic Resonance Center

College of Chemistry and Chemical Engineering

Lanzhou University

730000 Lanzhou, China

[1 Methods and Materials 3](#_Toc193872454)

[1.1 Materials 3](#_Toc193872455)

[1.2 Isotropic NMR Experiments and Assignment 3](#_Toc193872456)

[1.2.2 NOE Analysis 5](#_Toc193872457)

[1.2.3 Determination Amide Proton Temperature Coefficient 6](#_Toc193872458)

[1.3 Anisotropic NMR Experiments 9](#_Toc193872459)

[1.4 Computational Methods 12](#_Toc193872460)

[1.4.1 Generation of Structural Ensemble 12](#_Toc193872461)

[1.4.2 DFT Calculations 17](#_Toc193872462)

[1.4.2.1 Ensemble Refinement and Geometry Optimization 17](#_Toc193872463)

[1.4.2.2 NMR Calculations 21](#_Toc193872464)

[1.4.2.3 NMR Shift Referencing 21](#_Toc193872465)

[1.4.3 Sorting of the Final DFT Optimized Ensemble 22](#_Toc193872466)

[1.5 Conformational Analysis Using Stereofitter 23](#_Toc193872467)

[1.5.1 Stereofitter Backbone Determination with RDCs and ΔΔRCSAs 23](#_Toc193872468)

[1.5.1.1 Backbone Determination using RDC 23](#_Toc193872469)

[1.5.1.2 Backbone Determination using ΔΔRCSA 26](#_Toc193872470)

[1.5.1.2 Backbone Determination using ΔΔRCSA and RDC 27](#_Toc193872471)

[1.5.2 Conformational Analysis using Chemical Shifts, *J*-Couplings and Sidechain RDCs 29](#_Toc193872472)

[1.5.2.1 Analysis using Backbone and Proline RDCs 29](#_Toc193872473)

[1.5.2.2 Chemical Shift Analysis 32](#_Toc193872474)

[1.5.2.3 *J*-Coupling Analysis 33](#_Toc193872475)

[2. NMR Spectra 34](#_Toc193872476)

[3. Cartesian Coordinates 46](#_Toc193872477)

[4. Literature 55](#_Toc193872478)

1 Methods and Materials

# 1.1 Materials

The cyclic peptide heterophyllin B (**1**) has been bought from MedChemExpress (99.88% purity) and used as delivered. Fresh deuterated methanol (MeOH-*d_3_* and MeOD-*d_4_*) ampules (deuteration purity 99.8%) ordered from Sigma-Aldrich have been used for preparation of the NMR samples. The alignment medium AAKLVFF has been synthesized by CS Biotech with a purity of 98.0% and used as delivered.

# 1.2 Isotropic NMR Experiments and Assignment

Standard 1D ^1^H-, ^2^H- and ^13^C-spectra as well as 2D ^1^H,^1^H-COSY, ^1^H,^1^H-TOCSY, ^1^H,^1^H-NOESY, ^1^H,^13^C-HMBC and ^1^H,^13^C-HMQC experiments have been performed at 300 K using a Bruker AVANCE III 600 MHz spectrometer equipped with a 5 mm TCI cryoprobe.

Utilizing these spectra the ^1^H- and ^13^C-signals for **1** have been fully assigned, however methylene protons have been assigned as pairs and not individually. *J*-coupling values have been extracted from the 1H-spectra.

**Table S1**. Assigned ^1^H and ^13^C chemical shifts for heterophyllin B (**1**) measured in MeOD-*d*_4_ at 300 K.

| Residue | Atom | Atom ID in XYZ files  (C/N-H) | d_C_ in [ppm] | d_H_ in [ppm] | ^1^H-^1^H *J-* Coupling  in [Hz] |
| --- | --- | --- | --- | --- | --- |
| 1Phe | Cα | 2-55 | 58.4 | 4.17 | 7.04 |
|  | N-H | 3-56 | – | 6.41 | d, 7.02 |
|  | Cβ | 11-(63,64) | 34.4 | 3.48, 3.25 |  |
|  | Cγ | 12 | 140.3 | – |  |
|  | Cδ | 13-65, 17-69 | 131.1 | 7.19 |  |
|  | Cε | 14-66, 16-68 | 129.5 | 7.31 |  |
|  | Cζ | 15-67 | 127.6 | 7.29 |  |
|  | CO | 1 | 174.7 | – |  |
| 2Ile | Cα | 48-98 | 62.3 | 3.87 | 11.35, 8.44 |
|  | N-H | 47-97 | – | 8.04 | 8.43 |
|  | Cβ | 50-99 | 36.3 | 1.93 | 11.35 |
|  | Cγ_1_ | 51-(100,101) | 27.2 | 1.45, 1.11 |  |
|  | Cγ_2_ | 53-(105,106,107) | 16.6 | 1.03 |  |
|  | Cδ | 52-(102,103,104) | 10.3 | 0.83 |  |
|  | CO | 49 | 175.6 | – |  |
| 3Pro | Cα | 41-90 | 62.5 | 4.11 |  |
|  | Cβ | 42-(91,92) | 31.9 | 2.52, 2.09 |  |
|  | Cγ | 43-(93,94) | 22.9 | 1.95, 1.58 |  |
|  | Cδ | 44-(95,96) | 47.7 | 3.46, 3.37 |  |
|  | CO | 45 | 171.9 | – |  |
| 4Pro | Cα | 34-83 | 60.1 | 3.20 |  |
|  | Cβ | 35-(84,85) | 29.2 | 2.01, 1.57 |  |
|  | Cγ | 36-(86,87) | 26.3 | 2.00, 1.74 |  |
|  | Cδ | 37-(88,89) | 48.8 | 3.61, 3.48 |  |
|  | CO | 38 | 172.7 | – |  |
| 5Pro | Cα | 27-76 | 60.6 | 4.53 |  |
|  | Cβ | 28-(77,78) | 28.3 | 2.40, 1.81 |  |
|  | Cγ | 29-(79,80) | 26.5 | 2.05, 1.94 |  |
|  | Cδ | 30-(81,82) | 49.1 | 3.72, 3.46 |  |
|  | CO | 31 | 172.5 | – |  |
| 6Leu | Cα | 21-71 | 51.5 | 4.73 | 9.37 |
|  | N-H | 20-70 | – | 7.38 | 9.36 |
|  | Cβ | 23-(72,114) | 39.7 | 2.01, 1.52 |  |
|  | Cγ | 24-74 | 27.0 | 1.69 |  |
|  | Cδ_1_ | 73-(111,112,113) | 23.7 | 0.97 |  |
|  | Cδ_2_ | 75-(108,109,110) | 21.7 | 0.98 |  |
|  | CO | 22 | 172.5 | – |  |
| 7Gly | Cα | 10-(61,62) | 43 | 4.43, 3.43 |  |
|  | N-H | 9-60 | – | 8.37 |  |
|  | CO | 18 | 171.6 | – |  |
| 8Gly | Cα | 6-(58,59) | 45.9 | 3.96, 3.70 |  |
|  | N-H | 5-57 | – | 8.58 |  |
|  | CO | 7 | 172.9 | – |  |

### 1.2.2 NOE Analysis

Key proton-proton distances have been determined using a ^1^H,^1^H-NOESY spectrum measured in MeOD-*d_4_* at 300 K, using a standard pulse program with a mixing time of 60 ms. From the spectra three key cross-peaks between hydrogens from different residues were observed. These are listed below together with the methylene cross-peak from 1Phe-Cβ used as a reference for the distance calculations. The distances have been calculated based on the assumption that the methylene hydrogen distance is fixed at 1.8 Å with,

$\frac{d_{x}}{d_{ref}}=\left( \frac{Int_{x}}{Int_{ref}} \right)^{-\frac{1}{6}}$,

where “*d”* is the distance and “*Int”* the intensity of the cross-peak as measured in Brukers TopSpin software.

**Table S2.** Distances derived from cross-peak intensities extracted from a ^1^H,^1^H-NOESY spectrum with a mixing time of 60 ms measured in MeOD-*d_4_* at 300 K.

| Atom 1 | Atom 2 | Relative Intensity | Distance in [Å] |
| --- | --- | --- | --- |
| 1Phe Hβ1 | 1Phe Hβ2 | 1.00 | 1.80 |
| 3Pro Hα | 4Pro Hα | 0.32 | 2.18 |
| 5Pro Hα | 4Pro Hδ1-δ2 | 0.22/0.36 | 2.22 |
| 6Leu Hα | 5Pro Hδ1/2 | 0.33 | 2.16 |

### 1.2.3 Determination Amide Proton Temperature Coefficient

Amide proton temperature coefficients have been measured following standard procedures.^[1]^ One dimensional ^1^H-spectra have been measured for **1** in MeOH-*d_3_* for a temperature range of 291 K to 315 K in steps of 3 K. The obtained chemical shifts for the five amide protons have been plotted together with a linear regression to obtain the amide temperature coefficients as the slope of the equation,

$\delta_{NH}\left( T \right)=\Delta\delta_{NH}\cdot T+C$,

in which $\delta_{NH}$is the amide proton chemical shift, $T$the temperature, $C$the y-axis intercept and $\Delta\delta_{NH}$ the amide proton temperature coefficient usually reported in [ppb/K]. Additionally, *R^2^* is shown in each plot to indicate the quality of the linear regression. These show that the assumption of a linear dependency is justified in this temperature range for most residues investigated. An exception is 1Phe where a comparably low *R^2^* can be explained with the overall small temperature coefficient resulting in a higher influence of the measurement error.


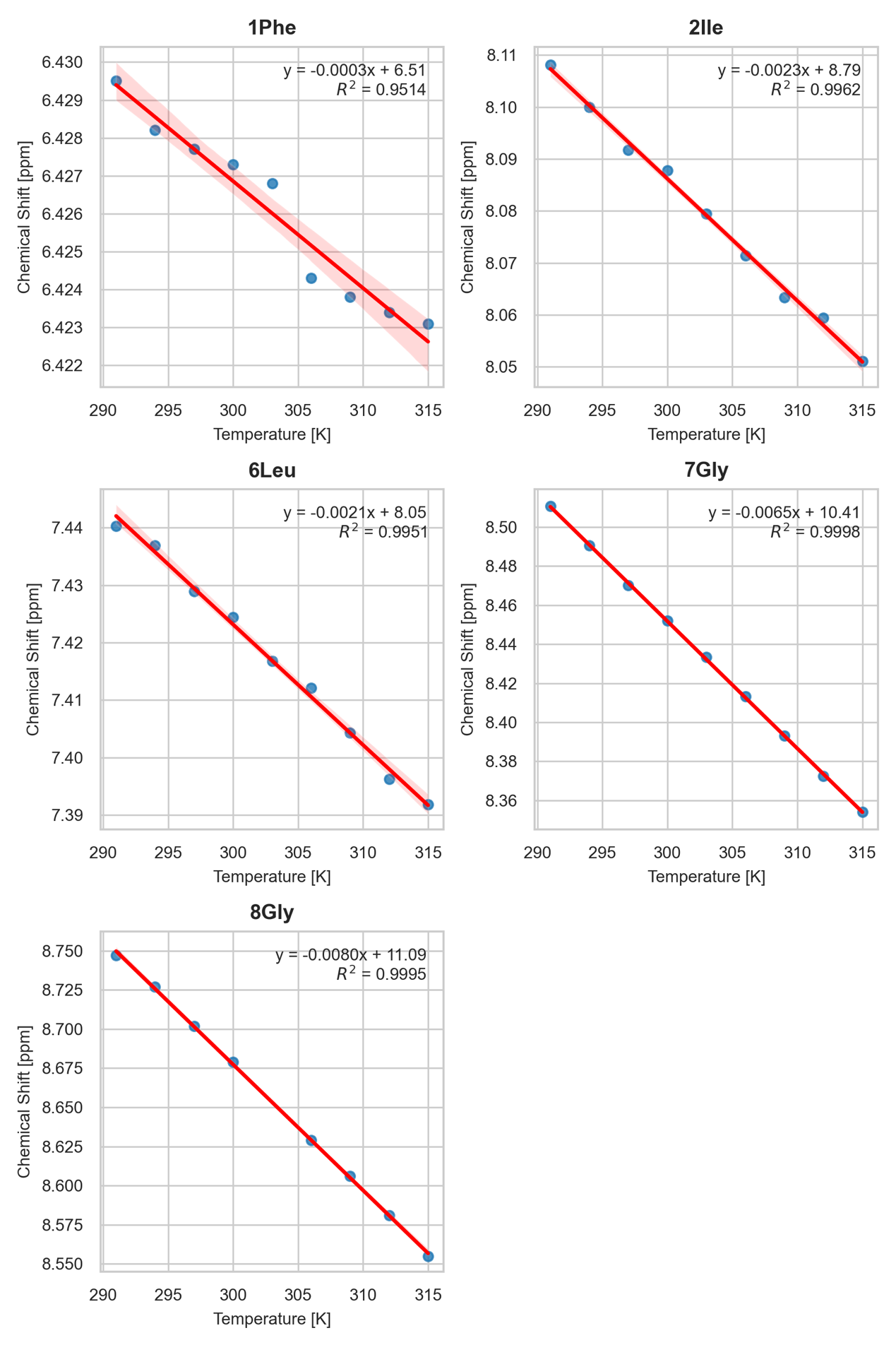


**Figure S1.** Chemical shifts of amide protons of **1** determined by ^1^H-spectra in MeOH-*d_3_* over the temperature range 291 K – 315 K. The red lines indicate a linear regression with the parameters shown in the upper right corner of each graph.

1.3 Anisotropic NMR Experiments

To measure the anisotropic NMR parameters RDCs and ΔΔRCSAs the previously described procedure^[2]^ has been adopted. F2-coupled ^1^H,^13^C-CLIP-HSQC and 1D-^13^C-spectra have been measured in methanol-*d_4_* under isotropic conditions. Following, the oligopeptide AAKLVFF has been added to obtain a (8 mg)/(350 mL) solution of AAKLVFF in MeOD-*d_4_*. Immediately afterwards another set of F2-coupled ^1^H,^13^C-CLIP-HSQC and 1D-^13^C-spectra have been measured. In addition, a 1D ^2^H-spectra has been measured to confirm, by looking at the strength of the quadrupolar splitting of the methanol signals, negligible alignment strength in this initial stage. The alignment process has been monitored by daily ^2^H-spectra until the quadrupolar splitting of the methanol signals reached a stationary value, see in Figure S2 a comparison between the initial and final quadrupolar splitting. The final splitting was 11.3 Hz for the OD-signal and 3.43 Hz for the CD_3_-signal of methanol. In the final alignment state F2-coupled ^1^H,^13^C-CLIP-HSQC and 1D-^13^C-spectra have been measured again.


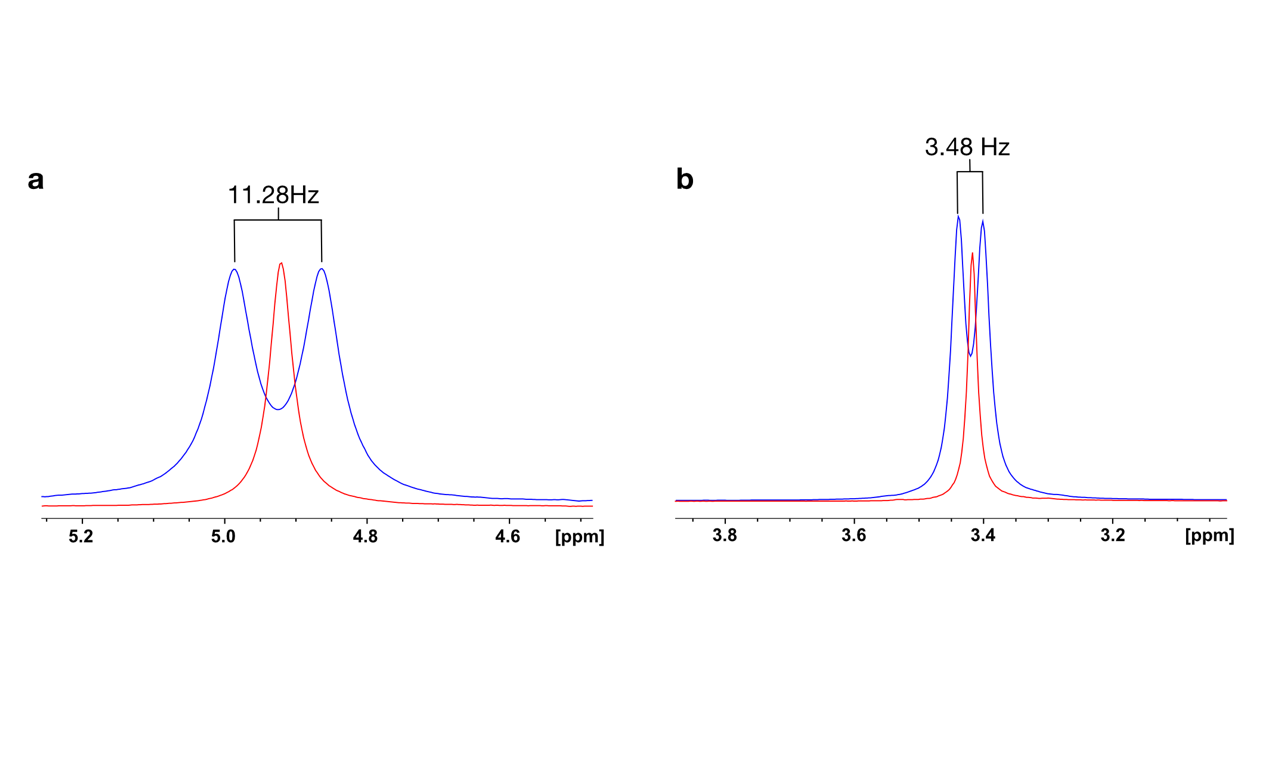
**Figure S2.** Quadrupolar ^2^H-splitting of the MeOD-*d_4_* signals observed in 1D ^2^H-spectra for (a) the -OD signal and (b) the -CD_3_ signal at 300 K.

From two F2-coupled ^1^H,^13^C-CLIP-HSQC under isotropic and final anisotropic conditions the ^1^*D*_C-H_ RDCs have been extracted following,

${{}^{1}D}_{CH} = {{}^{1}T}_{CH}-{{}^{1}J}_{CH}$,

where $T$is the C-H coupling constant in the final alignment stage and $J$is the C-H coupling constant under isotropic conditions. $D$denotes the measured RDC. Furthermore, ΔΔRCSA have been extracted using the equation,

$\Delta\Delta RCSA =\left( \delta_{K}^{final}-\delta_{Ref}^{final} \right)-\left( \delta_{K}^{inital}-\delta_{Ref}^{inital} \right)$,

in which $\delta_{K}$ is the carbon chemical shift of nucleus K and $\delta_{Ref}$ is the chemical shift of a reference carbon atom in the molecule. The difference is taken between the initial and final stage of alignment after addition of the alignment medium AAKLVFF.

**Table S3.** Measured anisotropic NMR data for heterophyllin B (**1**) in MeOD-*d_4_* with the alignment medium AAKLVFF at 300 K.

| Residue | Atom | RDC in [Hz] | ΔΔRCSA in [ppm]  Ref. Leu-Cγ | ΔΔRCSA in [ppm]  Ref. Ile-Cα |
| --- | --- | --- | --- | --- |
| 1Phe | Cα | 8.86 | 0.0152 | 0.0261 |
|  | NH |  |  |  |
|  | Cβ | -25.55; 7.75 | -0.0291 | -0.0182 |
|  | Cγ |  | -0.0427 | -0.0318 |
|  | Cδ | 4.92 | -0.0041 | 0.0068 |
|  | Cε | 4.31 | 0.001 | 0.0119 |
|  | Cζ | 16.36 | 0.0007 | 0.0116 |
|  | CO |  | 0.0459 | 0.0568 |
| 2Ile | Cα | 4.84 | -0.0109 | 0 |
|  | NH |  |  |  |
|  | Cβ | 6.06 | 0.0107 | 0.0216 |
|  | Cγ_1_ | 5.2; 7.52 | 0.0170 | 0.0279 |
|  | Cγ_2_ | -4.79 | 0.0137 | 0.0246 |
|  | Cδ | 0.98 | 0.041 | 0.0519 |
|  | CO |  | 0.0971 | 0.108 |
| 3Pro | Cα | -2.14 | 0.0183 | 0.0292 |
|  | Cβ | 15.32; 0.52 | 0.0345 | 0.0454 |
|  | Cγ | -9.32;16.26 | 0.0229 | 0.0338 |
|  | Cδ | 0.2; 8.14 | 0.0310 | 0.0419 |
|  | CO |  | 0.0261 | 0.037 |
| 4Pro | Cα | -0.23 | 0.0144 | 0.0253 |
|  | Cβ | -8.57; -4.4 | 0.0436 | 0.0545 |
|  | Cγ |  | 0.0649 | 0.0758 |
|  | Cδ | -7.29; -10.42 |  |  |
|  | CO |  | 0.0601 | 0.071 |
| 5Pro | Cα | -4.23 | 0.0317 | 0.0426 |
|  | Cβ | 18.8; -3.11 | 0.0091 | 0.02 |
|  | Cγ |  | 0.0586 | 0.0695 |
|  | Cδ | 6.97; 2.56 |  |  |
|  | CO |  |  |  |
| 6Leu | Cα | -7.13 | -0.0153 | -0.0044 |
|  | NH |  |  |  |
|  | Cβ | -9.19; -12.53 | -0.033 | -0.0221 |
|  | Cγ | -10.72 | 0.000 | 0.0109 |
|  | Cδ_1_ | -1.51 | 0.0647 | 0.0756 |
|  | Cδ_2_ | 1.56 | -0.0024 | 0.0085 |
|  | CO |  |  |  |
| 7Gly | Cα | 4.13; 16.97 | -0.022 | -0.0111 |
|  | NH |  |  |  |
|  | CO |  | 0.0820 | 0.0929 |
| 8Gly | Cα | -18.79; 3.85 | 0.0291 | 0.04 |
|  | NH |  |  |  |
|  | CO |  | 0.0525 | 0.0634 |

# 1.4 Computational Methods

## 1.4.1 Generation of Structural Ensemble

To sample the conformational space of the cyclic peptide **1** the *Conformer-Rotamer Ensemble Sampling Tool* (CREST) version 2.12^[3]^ has been used throughout in conjunction with the *extended Tight-Binding* (xTB) software package in version 6.6.1.^[4]^ The GFN-FF forcefield^[5]^ has been used for conformational exploration and subsequent optimization was done using the GFN2-xTB^[6]^ model. In the CREST approach the implicit solvation has been treated using the *Analytical-Linearized-Poisson-Boltzmann* (ALPB)^[7]^ model with parameters for methanol. The energy window has been set to include in the final CREST ensemble a 6 kcal/mol energy range based on GFN2-xTB/ALPB(methanol) free energies.

In initial tests, it was observed that the ensembles generated using CREST were in most cases comprised of conformers that retained the same cis/trans conformation of the three prolines compared to the starting structure. Therefore, all 8 possible combinations of cis/trans proline conformers were used as starting structures. From there, 8 conformational ensembles, depicted in Figures S3–S10*,* were obtained. As described in the main text some conformations were observed to be less stable and converted into other conformations during the sampling process. For these three tri-proline conformers, we performed additional CREST samplings while constraining the proline backbone atoms using a force constant of 0.5 E_h_/Bohr^2^. In all cases, the resulting ensembles consisted exclusively of conformers with the same backbone as the respective starting structure. The lowest-energy conformers from these ensembles were then used as starting structures for further unconstrained samplings. For the 3C-4C-5C and 3T-4C-5C conformers, the resulting ensembles remained similar to the newly defined starting structures. In the case of 3C-4C-5T, however, the new starting structure again converted into another conformer during the sampling, although the final ensemble still contained a small fraction of the original starting backbone conformation. It should be noted that the plots are labeled according to the initial structures, not the conformations they potentially converted into during the sampling process. Furthermore, a dihedral angle ψ > 90° indicates a trans conformation, while ψ < 90° indicated a cis conformation.


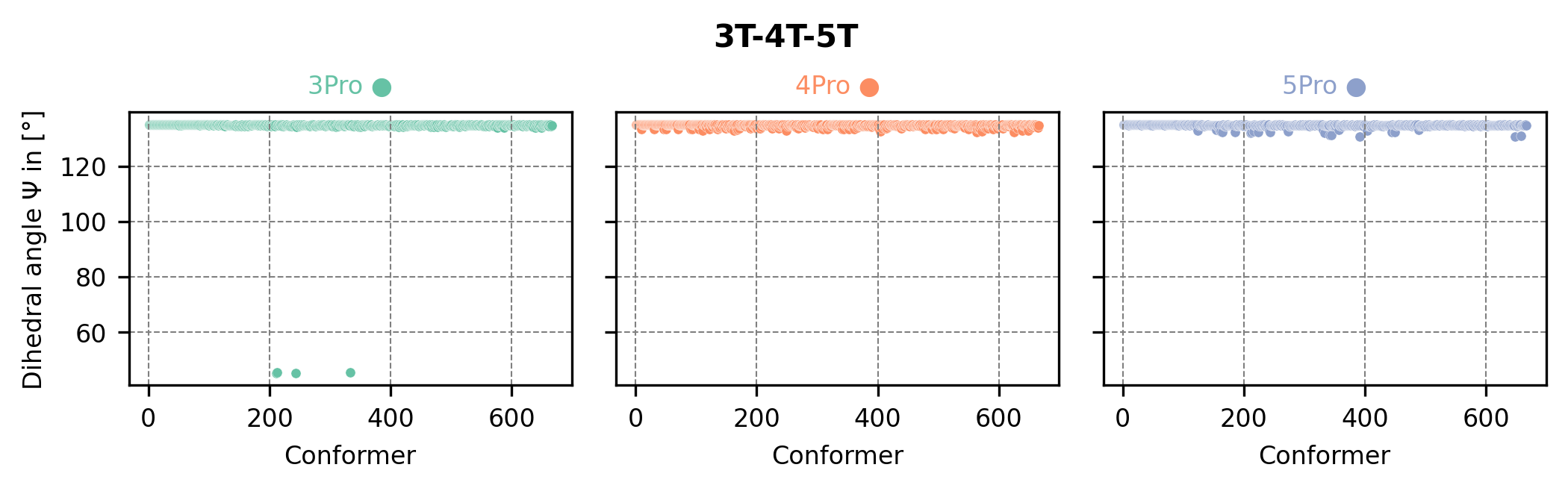
**Figure S3**. Dihedral angles of 3Pro, 4Pro and 5Pro for the CREST ensemble with the starting conformation TTT.


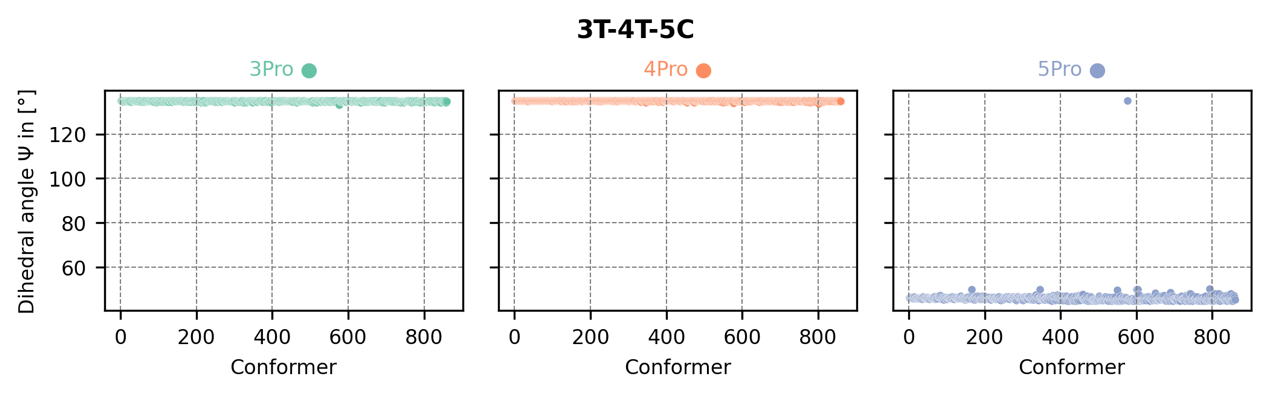


**Figure S4**. Dihedral angles of 3Pro, 4Pro and 5Pro for the CREST ensemble with the starting conformation TTC.


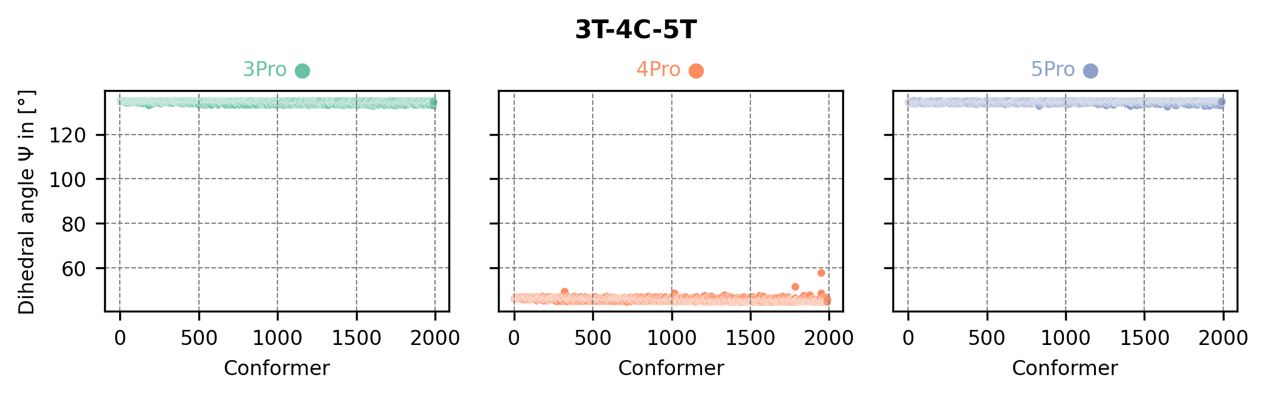
**Figure S5**. Dihedral angles of 3Pro, 4Pro and 5Pro for the CREST ensemble with the starting conformation TCT.


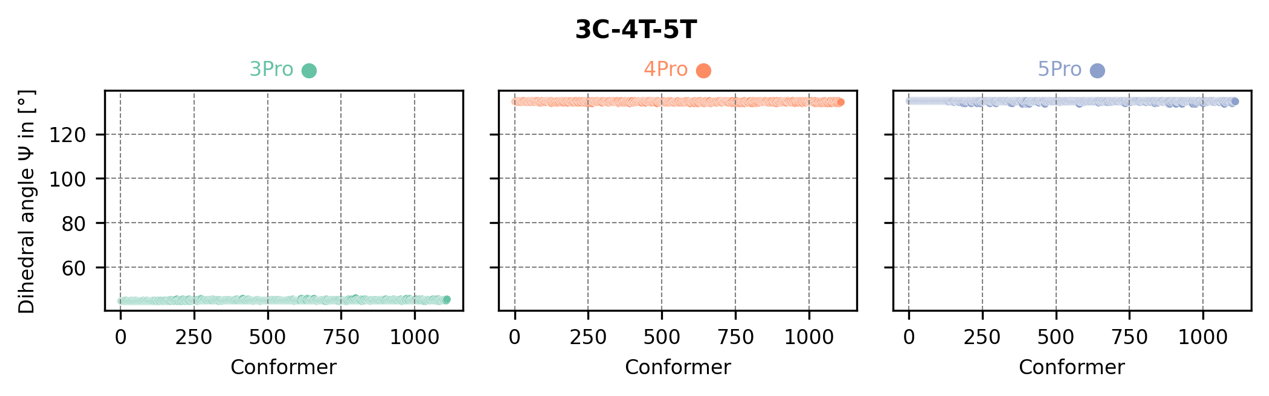
**Figure S6**. Dihedral angles of 3Pro, 4Pro and 5Pro for the CREST ensemble with the starting conformation CTT.


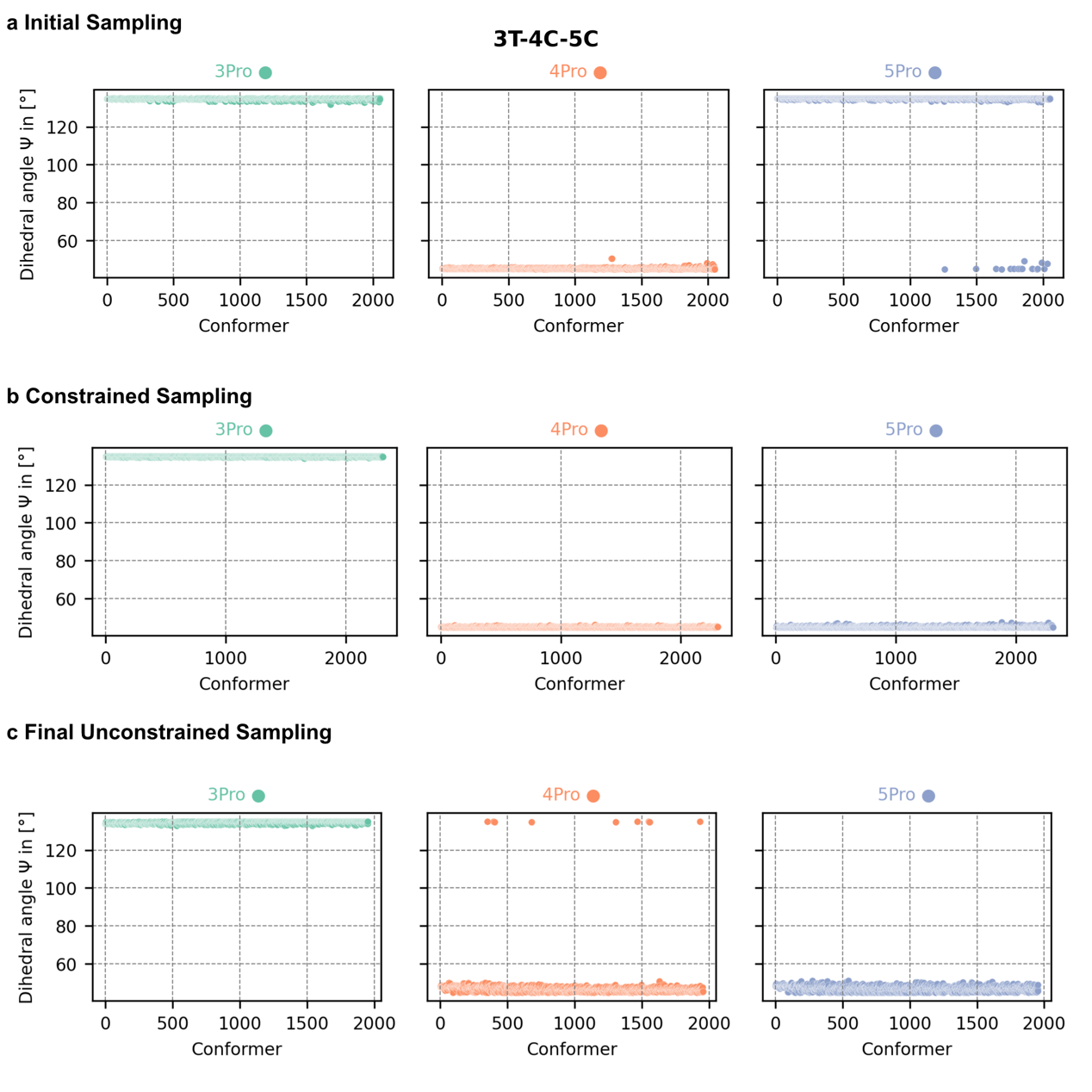
**Figure S7**. Dihedral angles of 3Pro, 4Pro and 5Pro for the CREST ensemble with the starting conformation TCC shown for (a) the initial sampling, (b) sampling with constrained proline backbone, and (c) unconstrainted sampling using the lowest energy conformer of (b) as a starting structure.


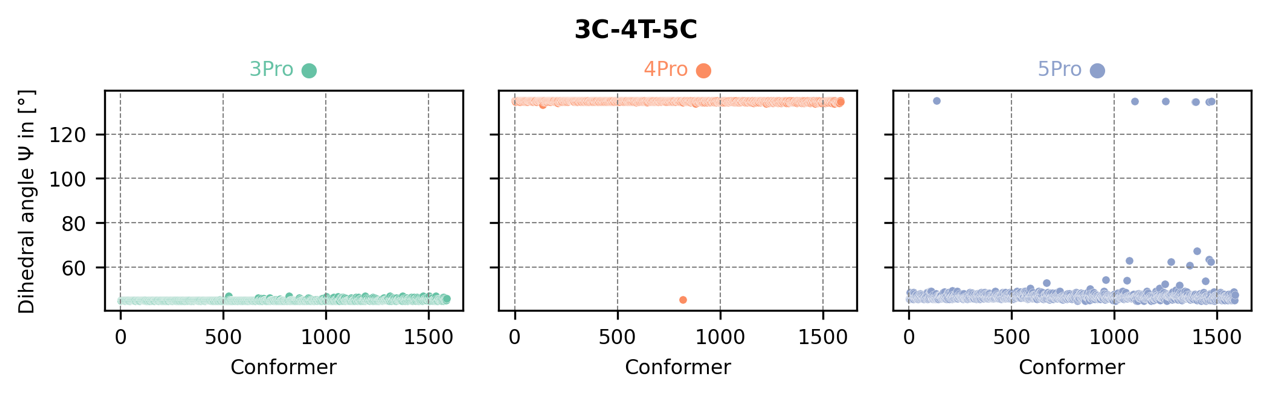


**Figure S8**. Dihedral angles of 3Pro, 4Pro and 5Pro for the CREST ensemble with the starting conformation CTC.


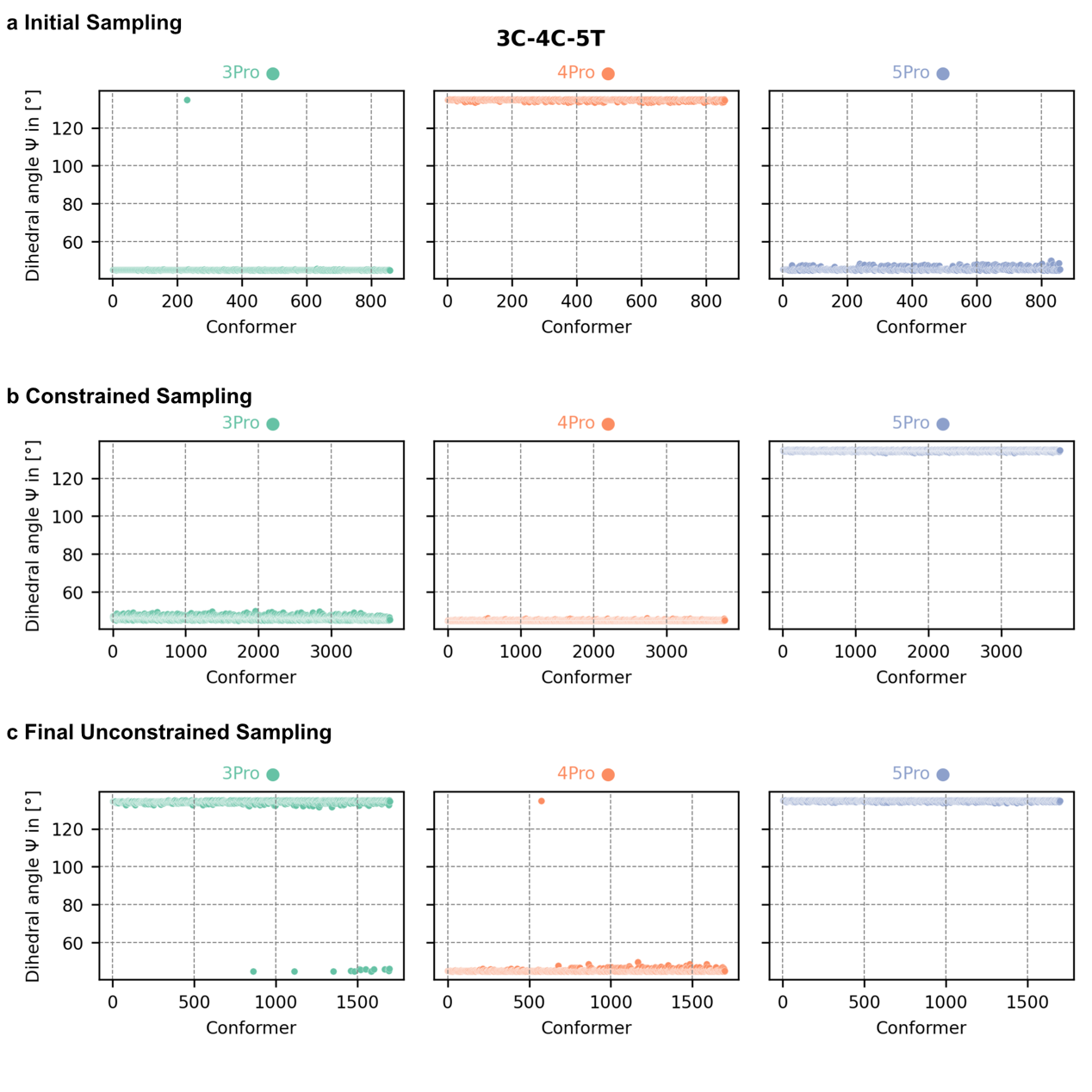


**Figure S9**. Dihedral angles of 3Pro, 4Pro and 5Pro for the CREST ensemble with the starting conformation CCT shown for (a) the initial sampling, (b) sampling with constrained proline backbone, and (c) unconstrainted sampling using the lowest energy conformer of (b) as a starting structure.


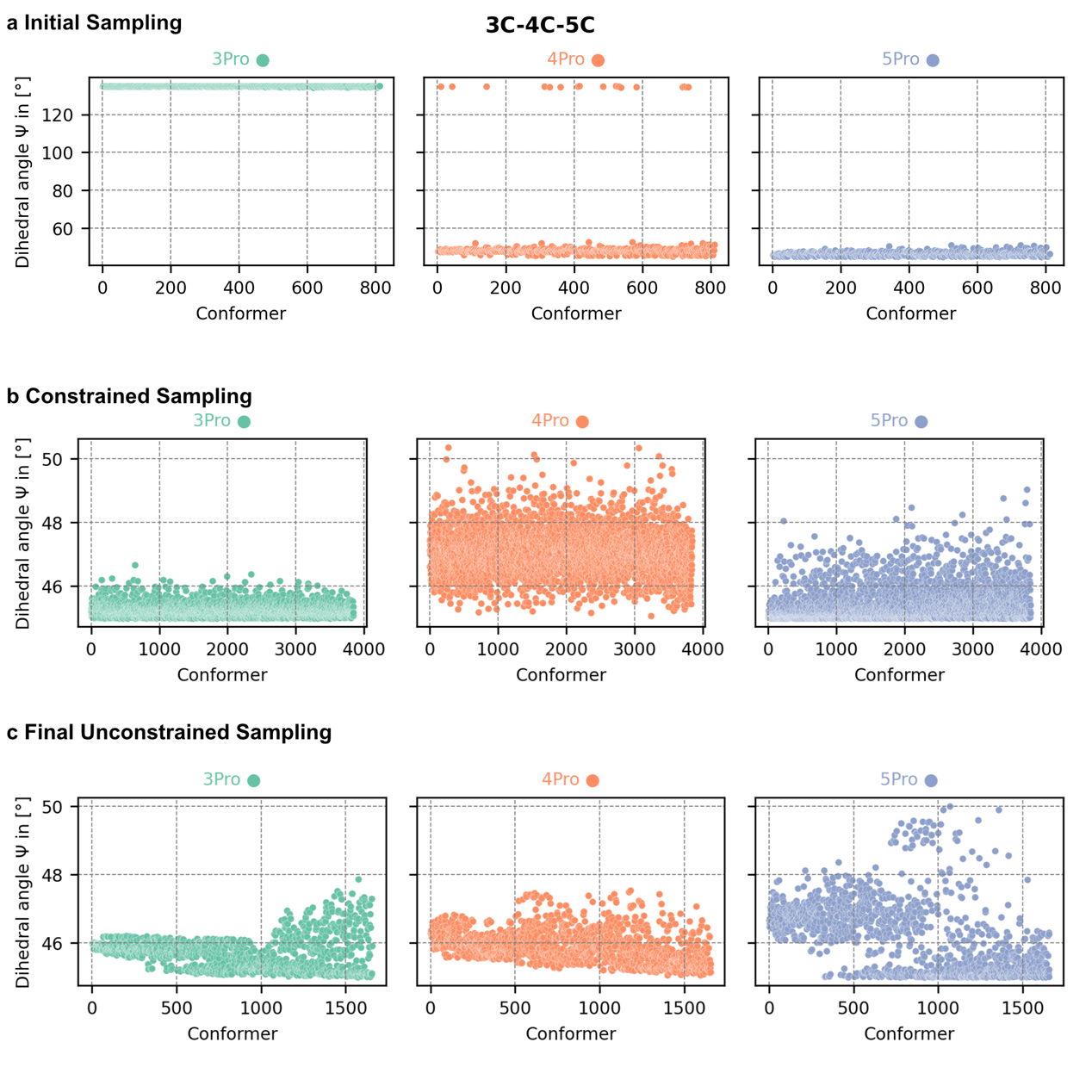


**Figure S10**. Dihedral angles of 3Pro, 4Pro and 5Pro for the CREST ensemble with the starting conformation CCC shown for (a) the initial sampling, (b) sampling with constrained proline backbone, and (c) unconstrainted sampling using the lowest energy conformer of (b) as a starting structure.

## 1.4.2 DFT Calculations

### 1.4.2.1 Ensemble Refinement and Geometry Optimization

As described in the main text, we only continued the ensemble refinement and DFT optimization with the ensemble obtained from the 3C-4T-5T CREST ensemble. For the ensemble refinement the *Commandline ENergetic SOrting* (CENSO) tool has been used. The refinement started with the 1474 conformers generated by CREST and refined it in a multistep process to only 13 energetically low conformers.

For all DFT calculations in the CENSO approach ORCA 5.0.4^[8–10]^ has been used. In the first part of CENSO the method PBE-D4/def2-SV(P)^[11–13]^ with SMD(methanol)^[14]^ solvation has been used. All conformers with a single-point energy difference from the lowest conformer above 6.0 kcal/mol have been discarded in this step. Afterwards, the same has been done with the method PW6B95-D4/def2-TZVP^[15]^ and a Gibbs free energy threshold of 5 kcal/mol, calculated using a *modified Rigid-Rotor-Harmonic-Oscillator* (mRRHO) approximation^[16]^ employing xTB-GFN2. The remaining conformers were DFT optimized at the PW6B95-D4/def2-TZVP/SMD(methanol) level of theory. Finally, the lowest energy ensemble whose conformers had a cumulative Boltzmann weight of 95% has been used for further investigation.

**Table S4.** Energies of the final DFT optimized conformers for heterophyllin B (**1**) using PW6B95-D4/def2-TZVP/SMD(methanol). Free energies have been calculated employing the mRRHO correction using xTB-GFN2.

| Conformers | Electronic energy [Eh] | MRRHO correction  [Eh] | Gibbs free energy  [Eh] | Relative Gibbs free energy  [kcal/mol] | Boltzmann weight  [%] |
| --- | --- | --- | --- | --- | --- |
| CONF2 | -2603.6274 | 0.8642 | -2602.7632 | 4.51 | 0.01 |
| CONF20 (I) | -2603.6309 | 0.8619 | -2602.7689 | 0.90 | 4.95 |
| CONF21 (II) | -2603.6311 | 0.8623 | -2602.7688 | 1.00 | 4.17 |
| CONF24 (III) | -2603.6304 | 0.8623 | -2602.7681 | 1.44 | 2.00 |
| CONF32 | -2603.6320 | 0.8616 | -2602.7704 | 0.00 | 22.73 |
| CONF33 | -2603.6309 | 0.8625 | -2602.7684 | 1.22 | 2.88 |
| CONF35 | -2603.6289 | 0.8626 | -2602.7663 | 2.59 | 0.29 |
| CONF37 | -2603.6287 | 0.8630 | -2602.7657 | 2.93 | 0.16 |
| CONF38 | -2603.6320 | 0.8621 | -2602.7700 | 0.25 | 14.82 |
| CONF40 | -2603.6320 | 0.8620 | -2602.7700 | 0.23 | 15.34 |
| CONF43 | -2603.6291 | 0.8630 | -2602.7662 | 2.65 | 0.26 |
| CONF44 | -2603.6295 | 0.8628 | -2602.7666 | 2.36 | 0.42 |
| CONF61 (IV) | -2603.6304 | 0.8628 | -2602.7676 | 1.77 | 1.15 |
| CONF62 (V) | -2603.6306 | 0.8626 | -2602.7680 | 1.52 | 1.75 |
| CONF64 (VI) | -2603.6301 | 0.8624 | -2602.7677 | 1.70 | 1.30 |
| CONF75 | -2603.6316 | 0.8620 | -2602.7696 | 0.48 | 10.19 |
| CONF77 | -2603.6274 | 0.8625 | -2602.7648 | 3.48 | 0.06 |
| CONF82 | -2603.6322 | 0.8625 | -2602.7697 | 0.43 | 10.99 |
| CONF112 | -2603.6245 | 0.8610 | -2602.7636 | 4.28 | 0.02 |
| CONF128 | -2603.6291 | 0.8627 | -2602.7665 | 2.47 | 0.35 |
| CONF130 | -2603.6271 | 0.8627 | -2602.7645 | 3.72 | 0.04 |
| CONF131 | -2603.6251 | 0.8611 | -2602.7641 | 3.97 | 0.03 |
| CONF137 | -2603.6267 | 0.8609 | -2602.7657 | 2.92 | 0.17 |
| CONF143 | -2603.6267 | 0.8609 | -2602.7658 | 2.90 | 0.17 |
| CONF148 | -2603.6257 | 0.8614 | -2602.7643 | 3.83 | 0.04 |
| CONF153 | -2603.6267 | 0.8629 | -2602.7638 | 4.11 | 0.02 |
| CONF172 | -2603.6246 | 0.8616 | -2602.7630 | 4.62 | 0.01 |
| CONF173 | -2603.6246 | 0.8615 | -2602.7631 | 4.59 | 0.01 |
| CONF197 | -2603.6261 | 0.8617 | -2602.7644 | 3.76 | 0.04 |
| CONF198 | -2603.6261 | 0.8617 | -2602.7644 | 3.76 | 0.04 |
| CONF201 | -2603.6291 | 0.8620 | -2602.7671 | 2.03 | 0.74 |
| CONF208 | -2603.6262 | 0.8613 | -2602.7650 | 3.40 | 0.07 |
| CONF212 | -2603.6263 | 0.8622 | -2602.7641 | 3.95 | 0.03 |
| CONF214 | -2603.6277 | 0.8639 | -2602.7638 | 4.13 | 0.02 |
| CONF216 | -2603.6242 | 0.8614 | -2602.7628 | 4.73 | 0.01 |
| CONF237 | -2603.6284 | 0.8630 | -2602.7654 | 3.12 | 0.12 |
| CONF238 | -2603.6271 | 0.8616 | -2602.7656 | 3.01 | 0.14 |
| CONF251 | -2603.6260 | 0.8614 | -2602.7646 | 3.65 | 0.05 |
| CONF253 | -2603.6284 | 0.8626 | -2602.7658 | 2.85 | 0.18 |
| CONF260 | -2603.6284 | 0.8626 | -2602.7659 | 2.85 | 0.19 |
| CONF264 | -2603.6262 | 0.8625 | -2602.7637 | 4.17 | 0.02 |
| CONF291 | -2603.6270 | 0.8617 | -2602.7653 | 3.18 | 0.11 |
| CONF304 | -2603.6280 | 0.8623 | -2602.7657 | 2.94 | 0.16 |
| CONF328 | -2603.6259 | 0.8618 | -2602.7641 | 3.92 | 0.03 |
| CONF357 | -2603.6284 | 0.8624 | -2602.7660 | 2.72 | 0.23 |
| CONF366 | -2603.6293 | 0.8611 | -2602.7682 | 1.37 | 2.24 |
| CONF420 | -2603.6243 | 0.8609 | -2602.7634 | 4.40 | 0.01 |
| CONF433 | -2603.6243 | 0.8610 | -2602.7634 | 4.41 | 0.01 |
| CONF446 | -2603.6284 | 0.8620 | -2602.7664 | 2.53 | 0.32 |
| CONF453 | -2603.6245 | 0.8617 | -2602.7628 | 4.74 | 0.01 |
| CONF515 | -2603.6258 | 0.8612 | -2602.7646 | 3.63 | 0.05 |
| CONF593 | -2603.6268 | 0.8628 | -2602.7640 | 4.00 | 0.03 |
| CONF626 | -2603.6287 | 0.8618 | -2602.7670 | 2.14 | 0.61 |
| CONF639 | -2603.6254 | 0.8611 | -2602.7642 | 3.87 | 0.03 |
| CONF660 | -2603.6253 | 0.8611 | -2602.7642 | 3.87 | 0.03 |
| CONF674 | -2603.6253 | 0.8611 | -2602.7642 | 3.88 | 0.03 |
| CONF815 | -2603.6232 | 0.8617 | -2602.7615 | 5.59 | 0.00 |
| CONF849 | -2603.6275 | 0.8626 | -2602.7649 | 3.46 | 0.07 |
| CONF1251 | -2603.6273 | 0.8625 | -2602.7648 | 3.51 | 0.06 |

### 1.4.2.2 NMR Calculations

The final conformational ensemble determined using CENSO has been used in GIAO-DFT calculations in ORCA 6.0.0 using the PW6B95/pcSseg-2/SMD(methanol) level of theory. Furthermore, a tight SCF criterion “TIGHTSCF” and the “RIJCOSX” with the auxiliary basis set def2/J has been used. For the treatment of the kinetic energy density $\tau$ in the exchange correlation functional of the PW6B95 functional the *Dobson* correction has been used.^[17]^ Chemical shielding tensors have been calculated for hydrogen-, carbon-, nitrogen- and oxygen-atoms and spin-spin coupling constants have been calculated for hydrogen- and carbon-atoms, using all contributions as originally described by Ramsey.^[18]^

All input and output files are deposited on Zenodo under the DOI: [10.5281/zenodo.14699304](https://doi.org/10.5281/zenodo.14699304).

### 1.4.2.3 NMR Shift Referencing

In order to compare the experimental chemical shifts with the calculated shielding constants, the DFT shielding constants have been converted to chemical shifts using the linear scaling approach:

$\delta_{calc}^{K}=a\cdot\sigma_{calc}^{K}+b$,

where $\sigma_{calc}^{K}$ are the DFT calculated shielding constants and $\delta_{calc}^{K}$ are the referenced calculated chemical shifts for nucleus K. $a$and $b$are the linear scaling parameters.

The linear scaling parameters for PW6B95-D4/pcSseg-2^[19]^/SMD(methanol) have been calculated using previously published experimental chemical shifts^[20]^ in methanol of a small set of small rigid molecules (acetone, acetonitrile, benzene, cyclohexane, cyclohexanone, DMF, DMAc, furane, nitromethane, pyridine, THF and toluene), which have been chosen based on their rigidity.^[21]^ The initial structures^[21]^ have been reoptimized with PW6B95-D4/def2-TZVP/SMD(methanol). These structures were then used in GIAO-DFT calculations with the same methods as described above for **1**.


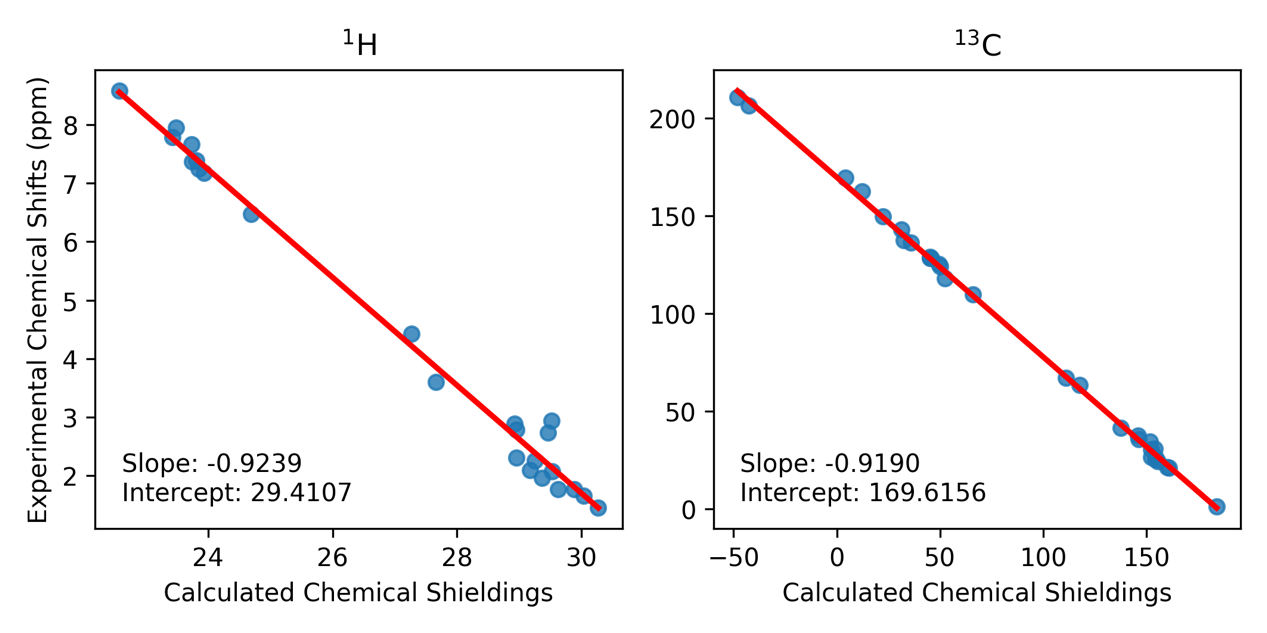
**Figure S11.** Determination of scaling parameters for hydrogen and carbon chemical shielding constants calculated using PW6B95/pcSseg-2/SMD(methanol) with structures optimized at the PW6B95-D4/def2-TZVP/SMD(methanol) level of theory.

## 1.4.3 Sorting of the Final DFT Optimized Ensemble

As mentioned in the main text, conformers with a cumulative Boltzmann weighting of 95% from the CENSO procedure were selected as the ensemble for further analysis using RDCs, ΔΔRCSAs, chemical shifts and *J*-couplings. However, visual inspection revealed that most of the conformers were very similar, prompting the addition of a clustering step.

Using a python script based on the PyMOL python interface, the conformers were grouped such that each member within a group differed from the others by a backbone RMSD of no more than 0.1 Å.

**Table S5.** Conformers obtained through the CENSO approach and clustered into groups of structures with backbone RMSD < 0.1 Å.

| Group | Conformer Name |
| --- | --- |
| 1 | CONF20 (**I**) |
|  | CONF21 (**II**) |
|  | CONF24 (**III**) |
|  | CONF61 (**IV**) |
|  | CONF62 (**V**) |
|  | CONF64 (**VI**) |
| 2 | CONF32 |
|  | CONF33 |
|  | CONF38 |
|  | CONF40 |
|  | CONF75 |
|  | CONF82 |
| 3 | CONF366 |

# 1.5 Conformational Analysis Using Stereofitter

## 1.5.1 Stereofitter Backbone Determination with RDCs and ΔΔRCSAs

Mestrelab Stereofitter 1.1.7 has been used in the MNOVA 15.1 software suite for all following analysis steps. As structural input files the output files from the ORCA NMR calculations have been used directly. In all calculations the conformers have been superimposed based on their backbone atoms, namely on the C_α_, CO and N atoms. These correspond to the following atom numbers (1,2,3,49,48,47,45,41,40,38,34,33,31,27,26,22,21,20,18,10,9,7,6,5). RDC errors have been estimated with 1.2 Hz and ΔΔRCSA errors with 0.005 Hz.

### 1.5.1.1 Backbone Determination using RDC

In the following Table S6 the experimental RDCs are summarized, which were used for the determination of the backbone conformation.

**Table S6.** RDC values that were used for the determination of the backbone conformation of **1**. The corresponding residues as well as the atom numbers, according to the cartesian coordinate files are shown.

| Residue | Carbon Number | Hydrogen Number | RDC in [Hz] |
| --- | --- | --- | --- |
| 1Phe | 2 | 55 | 8.86 |
| 2Ile | 48 | 98 | 4.84 |
| 3Pro | 41 | 90 | -2.14 |
| 4Pro | 34 | 83 | -0.23 |
| 5Pro | 27 | 76 | -4.23 |
| 6Leu | 21 | 71 | -7.13 |
| 7Gly | (10,61) | (10,62) | 10.55 |
| 8Gly | (6,58) | (6,59) | -7.47 |

First a single conformer approximation has been used. The resulting AIC and *Q*-factors are shown in Table S7.

**Table S7.** Results of RDC analysis in Stereofitter using only backbone RDC of **1** and the single conformer approximation.

| Conformer Name | Group | AIC Value | *Q*-factor |
| --- | --- | --- | --- |
| CONF62 (V) | G1 | 5.82 | 0.1565 |
| CONF61 (IV) | G1 | 6.97 | 0.1712 |
| CONF21 (II) | G1 | 9.81 | 0.2031 |
| CONF24 (III) | G1 | 10.27 | 0.2078 |
| CONF20 (I) | G1 | 11.24 | 0.2174 |
| CONF64 (VI) | G1 | 12.50 | 0.2292 |
| CONF82 | G2 | 39.22 | 0.4062 |
| CONF75 | G2 | 39.97 | 0.4100 |
| CONF40 | G2 | 41.16 | 0.4161 |
| CONF38 | G2 | 42.00 | 0.4203 |
| CONF33 | G2 | 42.13 | 0.4210 |
| CONF32 | G2 | 44.77 | 0.4339 |
| CONF366 | G3 | 47.43 | 0.4466 |

Using the multiple conformer single tensor (MCST) approach in Stereofitter did not improve the results, see Table S8. CONF62 was still the best even compared to ensembles with multiple conformers. Ensembles with up to three conformers were set to be tested, however only single conformers and ensembles with a maximum of 2 conformers were under the ensembles with the 10 lowest AICs. Furthermore, all ensembles showed very high (80-100%) weights for the conformers CONF62 or CONF61.

**Table S8.** Results of RDC analysis in Stereofitter using only backbone RDC of **1** and the multiple conformer approximation. Only the 10 ensembles with the lowest AIC are shown and only ensembles with up to three conformers were tested by Strereofitter.

| Conformer 1 Name | Conformer 2 Name | Weights | AIC Value | *Q*-factor |
| --- | --- | --- | --- | --- |
| CONF62 (V) | – | 1.00/0.00 | 5.82 | 0.1565 |
| CONF61 (IV) | – | 1.00/0.00 | 6.97 | 0.1712 |
| CONF62 (V) | CONF33 | 0.93/0.07 | 7.59 | 0.1533 |
| CONF61 (IV) | CONF33 | 0.86/0.14 | 7.75 | 0.1555 |
| CONF62 (V) | CONF366 | 0.95/0.05 | 7.78 | 0.1559 |
| CONF62 (V) | CONF82 | 0.97/0.03 | 7.79 | 0.1561 |
| CONF62 (V) | CONF75 | 0.99/0.01 | 7.81 | 0.1564 |
| CONF62 (V) | CONF38 | 0.99/0.01 | 7.82 | 0.1564 |
| CONF62 (V) | CONF40 | 0.99/0.01 | 7.82 | 0.1564 |
| CONF64 (VI) | CONF33 | 0.73/0.27 | 8.16 | 0.1609 |

### 1.5.1.2 Backbone Determination using ΔΔRCSA

Although one-bond C-H RDCs were sufficient to establish the main conformation of compound **1**, we also tested the use of ΔΔRCSAs for determining its backbone conformation. Table S9 summarizes the experimental ΔΔRCSA values that were used in the fitting procedure.

**Table S9.** ΔΔRCSAs used for the determination of the backbone conformation of **1**. The corresponding residues as well as the atom numbers, according to the XYZ coordinate files are shown.

| Residue | Carbon Number | ΔΔRCSA in [ppm] with Ref. 2Ile-Cα |
| --- | --- | --- |
| 1Phe | 2 | 0.0261 |
| 2Ile | 48 | 0.000 |
| 3Pro | 41 | 0.0292 |
| 4Pro | 34 | 0.0253 |
| 5Pro | 27 | 0.0426 |
| 6Leu | 21 | -0.004 |
| 7Gly | 10 | -0.011 |
| 8Gly | 6 | 0.0400 |

Using the experimental data shown above, reasonable results were obtained (Table S10). Similar to the results from the RDC analysis, all conformers of G1 exhibit lower AICs and *Q*-factors compared to those of G2 or G3. However, the discrimination between the groups is less pronounced than in the RDC analysis. Additionally, different referencing carbon atoms have been tried.

**Table S10.** Results obtained in the conformational analysis using the experimental backbone ΔΔRCSAs together with the chemical shielding tensors, calculated at the PW6B95/pcSseg-2/SMD(methanol) level of theory, in Stereofitter.

| Conformer  Name | Group | AIC  Ref. 6Leu-Cγ | AIC  Ref. 2Ile-Cα | *Q*-factor  Ref. 2Ile-Cα |
| --- | --- | --- | --- | --- |
| CONF21 (II) | G1 | 13.73 | 6.41 | 0.1672 |
| CONF62 (V) | G1 | 13.81 | 19.82 | 0.2940 |
| CONF64 (VI) | G1 | 14.70 | 19.20 | 0.2894 |
| CONF20 (I) | G1 | 15.00 | 16.14 | 0.2654 |
| CONF24 (III) | G1 | 15.51 | 19.28 | 0.2900 |
| CONF61 (IV) | G1 | 16.07 | 18.88 | 0.2869 |
| CONF38 | G2 | 19.68 | 23.76 | 0.3220 |
| CONF40 | G2 | 19.70 | 23.73 | 0.3217 |
| CONF33 | G2 | 22.21 | 17.72 | 0.2780 |
| CONF32 | G2 | 22.71 | 24.38 | 0.3261 |
| CONF366 | G3 | 27.07 | 26.741 | 0.3415 |
| CONF82 | G2 | 23.85 | 27.431 | 0.3459 |
| CONF75 | G2 | 29.31 | 30.203 | 0.3630 |

### 1.5.1.2 Backbone Determination using ΔΔRCSA and RDC

Since reasonable results were obtained using both RDC and ΔΔRCSA data independently, we also combined the two datasets for a joint analysis.

**Table S11.** Experimental RDCs and ΔΔRCSAs used together for the determination of the backbone conformation of **1**. The corresponding residues as well as the atom numbers, according to the XYZ coordinate files are shown.

| Residue | Carbon Number | Hydrogen Number | RDC in [Hz] or ΔΔRCSA in [ppm] |
| --- | --- | --- | --- |
| 1Phe | 2 | 55 | 8.86 |
| 1Phe | 2 | – | 0.0261 |
| 2Ile | 48 | 98 | 4.84 |
| 2Ile | 48 | – | 0 |
| 3Pro | 41 | 90 | -2.14 |
| 3Pro | 41 | – | 0.0292 |
| 4Pro | 34 | 83 | -0.23 |
| 4Pro | 34 | – | 0.0253 |
| 5Pro | 27 | 76 | -4.23 |
| 5Pro | 27 | – | 0.0426 |
| 6Leu | 21 | 71 | -7.13 |
| 6Leu | 21 | – | -0.0044 |
| 7Gly | (10,61) | (10,62) | 10.55 |
| 7Gly | 10 | – | -0.0111 |
| 8Gly | (6,58) | (6,59) | -7.47 |
| 8Gly | 6 | – | 0.040 |

Using the data shown above, poor fitting results were obtained in the conformational analysis (Table S12). Therefore, this approach was not pursued further.

**Table S12.** Results of the combined ΔΔRCSA/RDC analysis in Stereofitter.

| Conformer  Name | Group | AIC  Ref. 2Ile-Cα | *Q*-factor  Ref. 2Ile-Cα |
| --- | --- | --- | --- |
| CONF61 (IV) | G1 | 126.92 | 0.70 |
| CONF64 (VI) | G1 | 129.40 | 0.68 |
| CONF62 (V) | G1 | 129.53 | 0.71 |
| CONF24 (III) | G1 | 132.35 | 0.70 |
| CONF20 (I) | G1 | 133.18 | 0.70 |
| CONF21 (II) | G1 | 163.68 | 0.79 |
| CONF366 | G3 | 174.49 | 0.70 |
| CONF75 | G2 | 188.98 | 0.77 |
| CONF32 | G2 | 202.59 | 0.79 |
| CONF82 | G2 | 207.64 | 0.82 |
| CONF40 | G2 | 211.12 | 0.82 |
| CONF33 | G2 | 214.57 | 0.83 |
| CONF38 | G2 | 215.69 | 0.83 |

## 1.5.2 Conformational Analysis using Chemical Shifts, *J*-Couplings and Sidechain RDCs

### 1.5.2.1 Analysis using Backbone and Proline RDCs

Table S13 summarizes the RDCs that were used for the determination of the proline side-chain conformation. The same general setting as described above have been used. However, because the backbone has already been identified above, only the 6 conformers of G1 have been tested further.

**Table S13.** Backbone and proline RDCs used for the determination of the conformation of **1**. The corresponding residues as well as the atom numbers, according to the XYZ coordinate files are shown.

| Residue | Carbon Number | Hydrogen Number | RDC in [Hz] |
| --- | --- | --- | --- |
| 1Phe | 2 | 55 | 8.86 |
| 2Ile | 48 | 98 | 4.84 |
| 3Pro | 41 | 90 | -2.14 |
|  | 42 | 92 | 15.32 |
|  | 42 | 91 | 0.52 |
|  | 43 | 94 | -9.32 |
|  | 43 | 93 | 16.26 |
|  | 44 | 96 | 0.2 |
|  | 44 | 95 | 8.14 |
| 4Pro | 34 | 83 | -0.23 |
|  | 35 | 85 | -8.57 |
|  | 35 | 84 | -4.40 |
|  | 37 | 88 | -7.29 |
|  | 37 | 89 | -10.42 |
| 5Pro | 27 | 76 | -4.23 |
|  | 28 | 78 | 18.8 |
|  | 28 | 77 | -3.11 |
|  | 30 | 81 | 6.97 |
|  | 30 | 82 | 2.56 |
| 6Leu | 21 | 71 | -7.13 |
| 7Gly | 10 | 61 | 4.13 |
|  | 10 | 62 | 16.97 |
| 8Gly | 6 | 58 | -18.79 |
|  | 6 | 59 | 3.85 |

These RDCs were used to determine a minimum conformational ensemble in Stereofitter (Table S14). While the conformers CONF21, CONF20, CONF24 yield reasonable results, with *Q*-factors in the range of 0.2, increasing the number of conformers (Table S15), clearly led to a better fit with the experimental RDCs. However, including more than 2 conformers did not further improve the results. Therefore, only the combinations with the lowest AIC values using a maximum of 2 conformers are shown.

**Table S14.** Results of backbone and proline RDC fitting used for the determination of the conformation of **1** on single-conformer analyses.

| Conformer Name | Group | AIC Value | *Q*-factor |
| --- | --- | --- | --- |
| CONF21 (II) | G1 | 73.61 | 0.22 |
| CONF20 (I) | G1 | 73.96 | 0.22 |
| CONF24 (III) | G1 | 76.16 | 0.23 |
| CONF64 (VI) | G1 | 154.58 | 0.32 |
| CONF62 (V) | G1 | 386.73 | 0.51 |
| CONF61 (IV) | G1 | 389.77 | 0.51 |

**Table S15.** Results of backbone and proline RDC fitting used for the determination of the conformation of **1** using the multiple-conformer approach.

| Conformer 1 | Conformer 2 | Weights | AIC Value | *Q*-factor |
| --- | --- | --- | --- | --- |
| CONF24 (III) | CONF64 (**VI**) | 0.72/0.28 | 56.64 | 0.194 |
| CONF21 (II) | CONF64 (**VI**) | 0.72/0.28 | 57.70 | 0.196 |
| CONF20 (I) | CONF64 (**VI**) | 0.72/0.28 | 57.75 | 0.196 |
| CONF21 (II) | CONF62 (**V**) | 0.85/0.15 | 61.11 | 0.201 |
| CONF20 (I) | CONF62 (**V**) | 0.85/0.15 | 61.21 | 0.201 |
| CONF21 (II) | CONF61 (**IV**) | 0.85/0.15 | 61.37 | 0.202 |
| CONF20 (I) | CONF61 (**IV**) | 0.85/0.15 | 61.46 | 0.202 |

As clearly shown by the results above, combining any conformer from the subgroup of CONF20, CONF21, CONF24 with CONF64 yields the best fit to the experimental data. However, combinations with CONF61 or CONF62 also produce reasonably good results. This suggests that the fitting of the RDCs of 4Pro benefits most from averaging of different pyrrolidine conformers. In contrast, 3Pro and 5Pro are already well-described by a single conformer and do not benefit significantly from averaging, because CONF24 and CONF64 have the same pyrrolidine conformer for 5Pro.

### 1.5.2.2 Chemical Shift Analysis

All chemical shifts measured have been used, however, unassigned methylene hydrogen signals have been used as averaged shifts. The DFT calculated shieldings have been converted to chemical shifts using the linear expression derived above. Furthermore, errors have been estimated for carbon chemical shifts to be 2 ppm and hydrogen 0.2 ppm.

**Table S16.** Results for the chemical shift analysis used for the determination of the conformation of **1** by using single conformers.

| Conformer Name | Group | AIC |
| --- | --- | --- |
| CONF62 (V) | G1 | 44.08 |
| CONF24 (III) | G1 | 50.05 |
| CONF20 (I) | G1 | 50.16 |
| CONF64 (VI) | G1 | 51.66 |
| CONF21 (II) | G1 | 55.04 |
| CONF61 (IV) | G1 | 60.83 |

**Table S17.** Results for **the** chemical shift analysis used for the determination of the conformation of **1** using multiple conformers in Stereofitter.

| Conformer 1 | Conformer 2 | Weights | AIC |
| --- | --- | --- | --- |
| CONF21 (II) | CONF62 (**V**) | 0.38/0.62 | 39.80 |
| CONF24 (III) | CONF62 (**V**) | 0.37/0.63 | 42.81 |
| CONF20 (I) | CONF62 (**V**) | 0.34/0.66 | 43.81 |
| CONF62 (V) | CONF61 (**VI**) | 0.68/0.32 | 44.03 |
| CONF21 (II) | CONF24 (**III**) | 0.44/0.56 | 44.37 |
| CONF21 (II) | CONF61 (**IV**) | 0.46/0.54 | 44.56 |
| CONF20 (I) | CONF21 (**II**) | 0.59/0.41 | 47.65 |
| CONF20 (I) | CONF24 (**III**) | 0.50/0.50 | 48.06 |
| CONF20 (I) | CONF61 (**VI**) | 0.54/0.46 | 48.10 |

As seen from the data, CONF62 shows the best agreement with the experimental chemical shift values. Furthermore, a slight improvement over the single-conformer fit with CONF62 is achieved when including approximately 40% of CONF20, CONF21 or CONF24 in the ensemble. A structural comparison of CONF62 and CONF21 reveals differences in the conformations of both 4Pro and 5Pro.

Using simple linear regression yields good agreement between experimental and computed chemical shifts for each conformer. The best ensemble (CONF21+CONF62) yields an R^2^ value of 0.99986, with a slope of 1.02 and a y-intercept of –0.28.

### 1.5.2.3 *J*-Coupling Analysis

4 coupling constants could be reliably extracted from the ^1^H-spectrum. These couplings were then used in Stereofitters fitting procedure and compared to the DFT calculated coupling constants for the 6 conformers of G1.

**Table 18.** *J*-coupling constants used in the conformational analysis of **1**. The corresponding residues as well as the atom numbers, according to the XYZ coordinate files are shown.

| Residue | Hydrogen A Number | Hydrogen B Number | *J*-Coupling in [Hz] |
| --- | --- | --- | --- |
| 1Phe | 55 | 56 | 7.04 |
| 2Ile | 98 | 99 | 11.35 |
|  | 98 | 97 | 8.44 |
| 6Leu | 70 | 71 | 9.37 |

The analysis yielded the AIC values summarized in Table S19. As can be seen from that table, all conformers gave very good agreement with the experimental data. Still, CONF62 gave the closet agreement. However, considering the overall good agreement no discrimination is possible based on these coupling constants.

**Table S19.** Results of *J*-coupling analysis in Stereofitter for a single conformer of **1**.

| Conformer  Name | AIC Value |
| --- | --- |
| CONF20 (I) | 0.50 |
| CONF21 (II) | 0.41 |
| CONF24 (III) | 0.48 |
| CONF61 (IV) | 0.60 |
| CONF62 (V) | 0.28 |
| CONF64 (VI) | 0.48 |

2. NMR Spectra

All NMR spectra used for this paper have been obtained as described above and are deposited at Zenodo as Bruker datasets. Processing of the raw FIDs has been done using Bruker Topspin 3.5pl7, while referencing and spectral assignments have been done using Mestrelab MNOVA 15.1. Assignments are shown by their numbering in the .xyz file for 3C-4T-5T.

**Figure S12.** Cα-region of the ^1^H NMR spectrum of compound **1** (600 MHz, MeOD-*d*_4_, 300 K). The H_α_ Signal of 6Leu overlaps with the solvent signal at 4.74 ppm.

**Figure S13.** NH-region of the ^1^H NMR spectrum of compound **1** (600 MHz, MeOH-*d*_3_, 300 K).

**Figure S14.** Cα-region ^13^C NMR spectrum of compound **1** (600 MHz, MeOD-*d*_4_, 300 K).

**Figure S15.** COSY spectrum of compound **1** (600 MHz, MeOD-*d*_4_, 300 K).

**Figure S16.** TOCSY spectrum of compound **1** (600 MHz, MeOD-*d*_4_, 300 K).

**Figure S17.** NOESY spectrum of compound **1** (600 MHz, MeOD-*d*_4_, 300 K).

**Figure S18.** HMQC spectrum of compound **1** (600 MHz, MeOD-*d*_4_, 300 K).

**Figure S19.** HMBC spectrum of compound **1** (600 MHz, MeOD-*d*_4_, 300 K).

**Figure S20.** CLIP-HSQC spectrum of compound **1** (600 MHz, MeOD-*d*_4_, 300 K). Aromatic Signals have been folded by using a spectral range for carbon of 0–80 ppm.

**Figure S21.** Anisotropic CLIP-HSQC spectrum of compound **1** (600 MHz, MeOD-*d*_4_, AAKLVFF, 300 K).

**Figure S22.** ^13^C in initial (red) and final (blue) anisotropic medium spectrum of compound **1** (600 MHz, MeOD-*d*_4_, AAKLVFF, 300 K).

3. Cartesian Coordinates

In the following the cartesian coordinates (.xyz) for the ensemble with the lowest *Q*-factor based on the RDC analysis including the proline sidechain RDCs (Table S15) are shown. This ensemble includes the conformers: CONF24 (**III**) and CONF64 (**VI**). The structures of all tested conformers are included in the CREST ensembles deposited on Zenodo.

**Table S20.** Cartesian coordinates of conformer CONF24 (**III**) optimized at the DFT level PW6B95/def2-TZVP/SMD(methanol).

| Number | Element | X in [Å] | Y in [Å] | Z in [Å] |
| --- | --- | --- | --- | --- |
| 1 | C | -14.370 | -26.844 | 10.057 |
| 2 | C | -0.0416 | -24.853 | 0.4196 |
| 3 | N | 0.5180 | -12.687 | 0.9546 |
| 4 | O | -21.170 | -17.284 | 13.646 |
| 5 | N | -19.242 | -39.268 | 10.643 |
| 6 | C | -32.118 | -41.604 | 16.655 |
| 7 | C | -43.689 | -34.843 | 0.9614 |
| 8 | O | -53.799 | -31.897 | 15.880 |
| 9 | N | -42.288 | -32.727 | -0.3509 |
| 10 | C | -52.201 | -26.079 | -11.484 |
| 11 | C | 0.8799 | -37.005 | 0.5786 |
| 12 | C | 22.752 | -34.210 | 0.1136 |
| 13 | C | 26.224 | -35.328 | -12.268 |
| 14 | C | 39.153 | -32.704 | -16.449 |
| 15 | C | 48.800 | -28.893 | -0.7267 |
| 16 | C | 45.402 | -27.635 | 0.6088 |
| 17 | C | 32.449 | -30.244 | 10.227 |
| 18 | C | -49.524 | -11.411 | -14.188 |
| 19 | O | -56.602 | -0.5399 | -22.266 |
| 20 | N | -39.562 | -0.5880 | -0.7410 |
| 21 | C | -34.906 | 0.7579 | -0.9418 |
| 22 | C | -21.582 | 0.7066 | -16.759 |
| 23 | C | -32.987 | 14.816 | 0.3964 |
| 24 | C | -45.612 | 20.920 | 10.005 |
| 25 | O | -14.922 | -0.3300 | -17.136 |
| 26 | N | -17.093 | 18.300 | -22.318 |
| 27 | C | -0.4027 | 18.317 | -28.642 |
| 28 | C | -0.2254 | 32.822 | -33.205 |
| 29 | C | -16.552 | 37.569 | -35.227 |
| 30 | C | -23.935 | 31.124 | -23.646 |
| 31 | C | 0.6927 | 14.511 | -18.942 |
| 32 | O | 0.5936 | 16.984 | -0.6882 |
| 33 | N | 18.027 | 0.9286 | -24.074 |
| 34 | C | 29.971 | 0.8352 | -15.833 |
| 35 | C | 40.909 | 0.4210 | -25.735 |
| 36 | C | 33.243 | -0.2390 | -37.076 |
| 37 | C | 20.638 | 0.5956 | -38.050 |
| 38 | C | 33.082 | 22.007 | -0.9904 |
| 39 | O | 30.405 | 32.230 | -16.192 |
| 40 | N | 39.087 | 22.513 | 0.1988 |
| 41 | C | 44.500 | 11.383 | 0.9673 |
| 42 | C | 56.209 | 17.889 | 16.935 |
| 43 | C | 50.700 | 31.679 | 20.179 |
| 44 | C | 43.247 | 35.443 | 0.7506 |
| 45 | C | 34.674 | 0.5391 | 19.672 |
| 46 | O | 38.835 | -0.1631 | 28.853 |
| 47 | N | 21.766 | 0.8065 | 17.847 |
| 48 | C | 11.545 | 0.3160 | 26.783 |
| 49 | C | 0.6618 | -10.639 | 22.656 |
| 50 | C | -0.0044 | 13.222 | 27.471 |
| 51 | C | 0.5135 | 26.790 | 32.178 |
| 52 | C | -0.5198 | 37.870 | 31.386 |
| 53 | C | -11.191 | 0.8136 | 36.434 |
| 54 | O | 0.3787 | -19.135 | 31.057 |
| 55 | H | -0.1918 | -23.093 | -0.6431 |
| 56 | H | 0.7269 | -0.5154 | 0.3206 |
| 57 | H | -13.561 | -47.152 | 0.8149 |
| 58 | H | -33.995 | -52.300 | 16.704 |
| 59 | H | -32.279 | -38.080 | 26.933 |
| 60 | H | -33.677 | -35.505 | -0.7908 |
| 61 | H | -53.049 | -30.972 | -21.140 |
| 62 | H | -61.840 | -26.815 | -0.6535 |
| 63 | H | 0.8949 | -40.085 | 16.201 |
| 64 | H | 0.4696 | -45.166 | -0.0124 |
| 65 | H | 18.769 | -38.398 | -19.464 |
| 66 | H | 41.724 | -33.705 | -26.883 |
| 67 | H | 58.901 | -26.925 | -10.515 |
| 68 | H | 52.836 | -24.609 | 13.307 |
| 69 | H | 29.816 | -29.260 | 20.661 |
| 70 | H | -34.277 | -11.586 | -0.0989 |
| 71 | H | -42.276 | 12.679 | -15.552 |
| 72 | H | -25.624 | 22.717 | 0.2626 |
| 73 | C | -56.947 | 10.919 | 11.592 |
| 74 | H | -42.742 | 24.343 | 19.969 |
| 75 | C | -50.240 | 33.044 | 0.2105 |
| 76 | H | -0.4123 | 11.541 | -37.127 |
| 77 | H | 0.2508 | 38.564 | -25.282 |
| 78 | H | 0.3896 | 33.487 | -42.109 |
| 79 | H | -17.461 | 48.379 | -35.158 |
| 80 | H | -20.482 | 33.820 | -44.652 |
| 81 | H | -34.499 | 29.721 | -25.632 |
| 82 | H | -22.831 | 36.999 | -14.540 |
| 83 | H | 28.462 | 0.0944 | -0.7983 |
| 84 | H | 45.973 | 13.113 | -29.399 |
| 85 | H | 48.273 | -0.2252 | -21.102 |
| 86 | H | 38.814 | -0.2521 | -46.384 |
| 87 | H | 30.660 | -12.613 | -34.441 |
| 88 | H | 12.335 | 0.0454 | -42.322 |
| 89 | H | 22.230 | 15.023 | -43.878 |
| 90 | H | 47.732 | 0.3297 | 0.3172 |
| 91 | H | 59.257 | 12.185 | 25.623 |
| 92 | H | 64.621 | 18.641 | 10.092 |
| 93 | H | 43.795 | 31.039 | 28.573 |
| 94 | H | 58.400 | 38.899 | 22.663 |
| 95 | H | 49.714 | 40.439 | 0.0319 |
| 96 | H | 34.598 | 41.775 | 0.9238 |
| 97 | H | 18.853 | 13.820 | 10.080 |
| 98 | H | 15.929 | 0.1966 | 36.652 |
| 99 | H | -0.3863 | 14.309 | 17.295 |
| 100 | H | 0.8715 | 25.771 | 42.440 |
| 101 | H | 13.763 | 29.657 | 26.186 |
| 102 | H | -0.0734 | 47.454 | 33.945 |
| 103 | H | -0.9262 | 38.703 | 21.314 |
| 104 | H | -13.513 | 36.238 | 38.197 |
| 105 | H | -15.180 | -0.1379 | 33.068 |
| 106 | H | -0.7602 | 0.6855 | 46.642 |
| 107 | H | -19.432 | 15.211 | 36.646 |
| 108 | H | -52.876 | 30.333 | -0.8114 |
| 109 | H | -42.491 | 40.672 | 0.1662 |
| 110 | H | -59.062 | 37.502 | 0.6664 |
| 111 | H | -53.688 | 0.1935 | 16.799 |
| 112 | H | -60.934 | 0.7919 | 0.1903 |
| 113 | H | -65.120 | 15.333 | 17.264 |
| 114 | H | -28.578 | 0.7655 | 10.867 |

**Table S21.** Cartesian coordinates of conformer CONF64 (**VI**) optimized at the DFT level PW6B95/def2-TZVP/SMD(methanol).

| Number | Element | X in [Å] | Y in [Å] | Z in [Å] |
| --- | --- | --- | --- | --- |
| 1 | C | -14.638 | -26.888 | 10.602 |
| 2 | C | -0.0712 | -25.093 | 0.4630 |
| 3 | N | 0.5032 | -12.901 | 0.9775 |
| 4 | O | -21.367 | -17.213 | 14.013 |
| 5 | N | -19.578 | -39.266 | 11.461 |
| 6 | C | -32.481 | -41.393 | 17.491 |
| 7 | C | -43.983 | -34.712 | 10.262 |
| 8 | O | -54.103 | -31.574 | 16.419 |
| 9 | N | -42.510 | -32.881 | -0.2894 |
| 10 | C | -52.328 | -26.308 | -11.046 |
| 11 | C | 0.8403 | -37.305 | 0.6314 |
| 12 | C | 22.281 | -34.723 | 0.1349 |
| 13 | C | 32.193 | -30.693 | 10.170 |
| 14 | C | 45.049 | -28.148 | 0.5701 |
| 15 | C | 48.138 | -29.580 | -0.7709 |
| 16 | C | 38.289 | -33.543 | -16.612 |
| 17 | C | 25.451 | -36.049 | -12.114 |
| 18 | C | -49.602 | -11.675 | -13.896 |
| 19 | O | -56.668 | -0.5725 | -22.031 |
| 20 | N | -39.615 | -0.6103 | -0.7188 |
| 21 | C | -34.949 | 0.7333 | -0.9323 |
| 22 | C | -21.666 | 0.6742 | -16.751 |
| 23 | C | -32.951 | 14.668 | 0.3997 |
| 24 | C | -45.544 | 20.807 | 10.075 |
| 25 | O | -15.037 | -0.3641 | -17.088 |
| 26 | N | -17.228 | 17.940 | -22.437 |
| 27 | C | -0.4187 | 18.024 | -28.837 |
| 28 | C | -0.2532 | 32.571 | -33.348 |
| 29 | C | -16.852 | 37.279 | -35.252 |
| 30 | C | -24.122 | 30.750 | -23.664 |
| 31 | C | 0.6804 | 14.185 | -19.168 |
| 32 | O | 0.5701 | 16.324 | -0.7060 |
| 33 | N | 17.982 | 0.9135 | -24.331 |
| 34 | C | 29.655 | 0.7791 | -15.791 |
| 35 | C | 40.522 | 0.2924 | -25.390 |
| 36 | C | 36.373 | 0.8961 | -38.705 |
| 37 | C | 21.260 | 0.7625 | -38.525 |
| 38 | C | 33.166 | 21.504 | -10.172 |
| 39 | O | 30.595 | 31.701 | -16.548 |
| 40 | N | 39.290 | 22.036 | 0.1665 |
| 41 | C | 44.561 | 10.849 | 0.9359 |
| 42 | C | 56.388 | 17.202 | 16.570 |
| 43 | C | 51.100 | 31.092 | 19.754 |
| 44 | C | 43.744 | 34.920 | 0.7046 |
| 45 | C | 34.707 | 0.4990 | 19.416 |
| 46 | O | 38.827 | -0.2144 | 28.529 |
| 47 | N | 21.826 | 0.7886 | 17.735 |
| 48 | C | 11.628 | 0.3126 | 26.778 |
| 49 | C | 0.6584 | -10.688 | 22.846 |
| 50 | C | 0.0104 | 13.264 | 27.431 |
| 51 | C | 0.5371 | 26.841 | 32.009 |
| 52 | C | -0.4892 | 37.975 | 31.066 |
| 53 | C | -11.049 | 0.8325 | 36.470 |
| 54 | O | 0.3755 | -19.054 | 31.378 |
| 55 | H | -0.2277 | -23.463 | -0.6010 |
| 56 | H | 0.7101 | -0.5456 | 0.3324 |
| 57 | H | -13.955 | -47.230 | 0.9099 |
| 58 | H | -34.424 | -52.074 | 17.769 |
| 59 | H | -32.646 | -37.644 | 27.688 |
| 60 | H | -33.896 | -35.801 | -0.7192 |
| 61 | H | -53.072 | -31.311 | -20.654 |
| 62 | H | -62.026 | -26.973 | -0.6205 |
| 63 | H | 0.8702 | -40.166 | 16.788 |
| 64 | H | 0.4118 | -45.541 | 0.0643 |
| 65 | H | 29.792 | -29.568 | 20.645 |
| 66 | H | 52.645 | -25.027 | 12.707 |
| 67 | H | 58.153 | -27.621 | -11.221 |
| 68 | H | 40.618 | -34.692 | -27.089 |
| 69 | H | 17.827 | -39.189 | -19.098 |
| 70 | H | -34.350 | -11.740 | -0.0692 |
| 71 | H | -42.353 | 12.394 | -15.448 |
| 72 | H | -25.597 | 22.561 | 0.2570 |
| 73 | C | -56.855 | 10.806 | 11.826 |
| 74 | H | -42.605 | 24.315 | 19.989 |
| 75 | C | -50.259 | 32.863 | 0.2122 |
| 76 | H | -0.4269 | 11.196 | -37.301 |
| 77 | H | 0.2286 | 38.274 | -25.427 |
| 78 | H | 0.3523 | 33.379 | -42.303 |
| 79 | H | -17.792 | 48.086 | -35.117 |
| 80 | H | -20.822 | 33.570 | -44.676 |
| 81 | H | -34.695 | 29.320 | -25.575 |
| 82 | H | -22.964 | 36.595 | -14.546 |
| 83 | H | 27.738 | 0.0675 | -0.7786 |
| 84 | H | 50.450 | 0.5891 | -22.157 |
| 85 | H | 40.190 | -0.7909 | -25.873 |
| 86 | H | 39.130 | 19.464 | -39.127 |
| 87 | H | 40.816 | 0.3875 | -47.195 |
| 88 | H | 18.081 | -0.2179 | -42.033 |
| 89 | H | 16.394 | 15.211 | -44.528 |
| 90 | H | 47.644 | 0.2715 | 0.2843 |
| 91 | H | 59.371 | 11.492 | 25.276 |
| 92 | H | 64.794 | 17.796 | 0.9703 |
| 93 | H | 44.155 | 30.595 | 28.127 |
| 94 | H | 58.910 | 38.195 | 22.233 |
| 95 | H | 50.333 | 39.704 | -0.0175 |
| 96 | H | 35.244 | 41.466 | 0.8718 |
| 97 | H | 18.922 | 13.702 | 10.014 |
| 98 | H | 16.061 | 0.2007 | 36.636 |
| 99 | H | -0.3746 | 14.293 | 17.262 |
| 100 | H | 0.8912 | 25.905 | 42.292 |
| 101 | H | 14.036 | 29.589 | 26.015 |
| 102 | H | -0.0377 | 47.563 | 33.518 |
| 103 | H | -0.8932 | 38.706 | 20.976 |
| 104 | H | -13.228 | 36.470 | 37.880 |
| 105 | H | -19.244 | 15.453 | 36.642 |
| 106 | H | -15.107 | -0.1193 | 33.201 |
| 107 | H | -0.7440 | 0.7114 | 46.679 |
| 108 | H | -42.535 | 40.509 | 0.1565 |
| 109 | H | -59.056 | 37.332 | 0.6718 |
| 110 | H | -52.971 | 30.072 | -0.8055 |
| 111 | H | -53.546 | 0.1868 | 17.081 |
| 112 | H | -60.907 | 0.7725 | 0.2190 |
| 113 | H | -64.992 | 15.257 | 17.521 |
| 114 | H | -28.494 | 0.7565 | 10.928 |

4. Literature

[1] T. Cierpicki, J. Otlewski, *J. Biomol. NMR* **2001**, *21*, 249–261.

[2] X.-L. Li, L.-P. Chi, A. Navarro-Vázquez, S. Hwang, P. Schmieder, X.-M. Li, X. Li,

S.-Q. Yang, X. Lei, B.-G. Wang, others, *J. Am. Chem. Soc.* **2019**, *142*, 2301–

2309.

[3] S. Grimme, *J. Chem. Theory Comput.* **2019**, *15*, 2847–2862.

[4] C. Bannwarth, E. Caldeweyher, S. Ehlert, A. Hansen, P. Pracht, J. Seibert, S.

Spicher, S. Grimme, *Wiley Interdiscip. Rev. Comput. Mol. Sci.* **2021**, *11*, e1493.

[5] S. Spicher, S. Grimme, *Angew. Chem., Int. Ed.* **2020**, *59*, 15665–15673.

[6] C. Bannwarth, S. Ehlert, S. Grimme, *J. Chem. Theory Comput.* **2019**, *15*, 1652–

1671.

[7] S. Ehlert, M. Stahn, S. Spicher, S. Grimme, *J. Chem. Theory Comput.* **2021**, *17*,

4250–4261.

[8] F. Neese, *Wiley Interdiscip. Rev. Comput. Mol. Sci*. **2022**, 12, e1606.

[9] F. Neese, F. Wennmohs, U. Becker, C. Riplinger, *J. Chem. Phys.* **2020**, *152*,

224108.

[10] F. Neese, *Wiley Interdiscip. Rev. Comput. Mol. Sci.* **2012**, 2, 73–78.

[11] C. Adamo, V. Barone, *J. Chem. Phys.* **1999**, *110*, 6158–6170.

[12] F. Weigend, R. Ahlrichs, *Phys. Chem. Chem. Phys.* **2005**, *7*, 3297–3305.

[13] E. Caldeweyher, J. M. Mewes, S. Ehlert, S. Grimme, *Phys. Chem. Chem. Phys.*

**2020**, *22*, 8499–8512.

[14] A. V. Marenich, C. J. Cramer, D. G. Truhlar, *J. Phys. Chem. B* **2009**, *113*, 6378–

6396.

[15] Y. Zhao, D. G. Truhlar, *J. Phys. Chem. A* **2005**, *109*, 5656–5667.

[16] S. Spicher, S. Grimme, *J. Chem. Theory Comput.* **2021**, *17*, 1701–1714.

[17] J. F. Dobson, *J. Chem. Phys.* **1993**, *98*, 8870–8872.

[18] N. F. Ramsey, *Phys. Rev.* **1953**, *91*, 303–307.

[19] F. Jensen, *J. Chem. Theory Comput.* **2015**, *11*, 132–138.

[20] G. R. Fulmer, A. J. M. Miller, N. H. Sherden, H. E. Gottlieb, A. Nudelman, B. M.

Stoltz, J. E. Bercaw, K. I. Goldberg, *Organometallics* **2010**, *29*, 2176–2179.

[21] R. D. Cohen, J. S. Wood, Y.-H. Lam, A. V Buevich, E. C. Sherer, M. Reibarkh,

R. T. Williamson, G. E. Martin, *Molecules* **2023**, *28*, 2449.
